# The TERB1-TERB2-MAJIN complex of mouse meiotic telomeres dates back to the common ancestor of metazoans

**DOI:** 10.1186/s12862-020-01612-9

**Published:** 2020-05-14

**Authors:** Irene da Cruz, Céline Brochier-Armanet, Ricardo Benavente

**Affiliations:** 1 Department of Cell and Developmental Biology, Biocenter, University of Würzburg, 97074 Würzburg, Germany; 2 Laboratoire de Biométrie et Biologie Evolutive, CNRS, UMR 5558, Université Lyon 1, F-69622 Villeurbanne, France

**Keywords:** Evolution, Meiosis, Metazoan, Telomere, TERB1, TERB2, MAJIN

## Abstract

**Background:**

Meiosis is essential for sexual reproduction and generates genetically diverse haploid gametes from a diploid germ cell. Reduction of ploidy depends on active chromosome movements during early meiotic prophase I. Chromosome movements require telomere attachment to the nuclear envelope. This attachment is mediated by telomere adaptor proteins. Telomere adaptor proteins have to date been identified in fission yeast and mice. In the mouse, they form a complex composed of the meiotic proteins TERB1, TERB2, and MAJIN. No sequence similarity was observed between these three mouse proteins and the adaptor proteins of fission yeast, raising the question of the evolutionary history and significance of this specific protein complex.

**Result:**

Here, we show the TERB1, TERB2, and MAJIN proteins are found throughout the Metazoa and even in early-branching non-bilateral phyla such as Cnidaria, Placozoa and Porifera. Metazoan TERB1, TERB2, and MAJIN showed comparable domain architecture across all clades. Furthermore, the protein domains involved in the formation of the complex as well as those involved for the interaction with the telomere shelterin protein and the LINC complexes revealed high sequence similarity. Finally, gene expression in the cnidarian *Hydra vulgaris* provided evidence that the TERB1-TERB2-MAJIN complex is selectively expressed in the germ line.

**Conclusion:**

Our results indicate that the TERB1-TERB2-MAJIN complex has an ancient origin in metazoans, suggesting conservation of meiotic functions.

## Background

Meiosis is a cell division mode during which one round of DNA replication is followed by two successive rounds of chromosome segregation leading, to the generation of haploid cells. During the prophase I stage, a series of specialized events including homologous chromosome pairing, synapsis, and recombination are crucial for the faithful completion of meiosis. These processes mainly depend on evolutionarily conserved chromosome movements and require the association of telomeres with the nuclear envelope (NE) [1, 2].

**Fig. 1:**
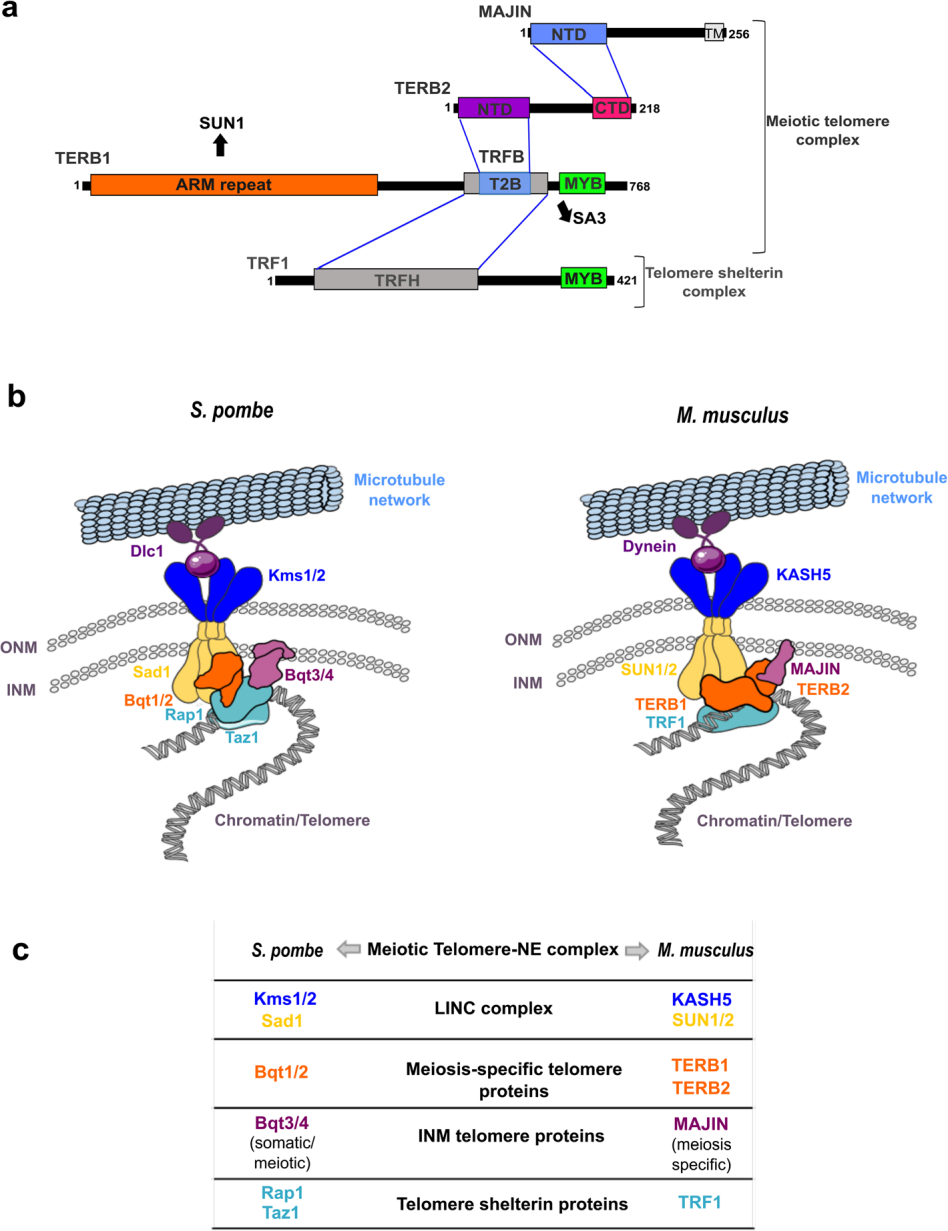
Meiosis telomere complex in *M. musculus* and S. *pombe.****a*** Schematic representation of the interacting domains mediating the simultaneous binding of TERB1-TERB2-MAJIN and telomeric shelterin protein TRF1. The MAJIN C-terminus contains a transmembrane binding domain (TM, aa 232–251) and interacts via its N-terminal domain (NTD, aa 1–120) with a specific C-terminal domain of TERB2 (CTD, aa 169–202). The TERB2 N-terminal domain (NTD, aa 2–194) interacts with the C-terminal domain of TERB1 (T2B, aa 593–622), encompassed by the TRF1-binding domain (TRFB, aa 523–699) which in turn interacts with the TRF homology domain of TRF1 (TRFH, aa 54–251). The TERB1 N-terminus contains an armadillo repeat (ARM repeat, aa 16–384) domain that is thought to interact with the SUN1/2. Both TERB1 and TRF1 also possess a MYB domain (MYB, aa 715–747; aa 367–416 respectively) that binds double-stranded telomeric DNA. The TERB1 MYB domain also interacts with the meiotic cohesin subunit SA3. **b** Molecular components involved in meiotic telomere attachment and movement through the nuclear envelope (NE) in *S. pombe* and *M. musculus*. The highly conserved LINC complex is composed of the SUN domain protein in the inner nuclear envelope (INM) and interacts in the perinuclear space with a KASH domain protein localized in the outer nuclear envelope (ONM). The LINC complex functions to connect the telomeres indirectly with cytoplasmic microtubules by the molecular linker dynein. Sad1-Kms1/2 and SUN1/2-KASH5 represents the LINC complex in *S. pombe* and *M. musculus* respectively. The association of telomeres to the NE requires meiosis-specific telomere adaptor proteins that bridge the interaction between the telomere sheltering complex and the LINC complex. In *S. pombe* shelterin-related proteins Rap1-Taz1 interact with meiosis-specific telomere protein Bqt1–2 to associate with Sad1. The ubiquitously expressed INM Bqt3–4 proteins also assist the interaction of telomeres to the NE through the association of Bqt4-Rap1. In mouse TERB1-TERB2-MAJIN meiotic telomere complex interact with TRF1 telomere shelterin protein and with SUN1/2. MAJIN protein has DNA binding properties and a single transmembrane domain similarly to the *S. pombe* Bqt4 protein. **c** Comparable components responsible of the meiotic telomere attachment and chromosome movements between *S. pombe* and *M. musculus*

The forces required for meiotic chromosome movements are generated in the cytoplasm and transduced into the nucleus via LINC (Linker of Nucleoskeleton and Cytoskeleton) complexes of the nuclear envelope (NE) [3–6]. In mice, this complex is composed of the transmembrane proteins SUN1 and SUN2 of the inner nuclear membrane (INM), and the KASH5 protein in the outer nuclear membrane (ONM) [7–11]. In addition, the meiotic telomere adaptor proteins TERB1, TERB2, and MAJIN [12–14] provide the physical linkage of telomeres to the NE. To accomplish this, TERB1 interacts with SUN1 through its N-terminal ARM repeat domain and with the meiotic cohesin subunit SA3, the telomere shelterin protein TRF1, and TERB2 through a region flanking its C-terminal MYB domain [13–17] (Fig. 1a). Simultaneously, a specific C-terminal region of TERB2 interacts with the MAJIN N-terminal domain [14, 18]. MAJIN possesses DNA binding properties and is anchored to the INM via a transmembrane helix at its C-terminus [13, 14, 19] (Fig. 1a).

The conserved movement of homologous chromosomes is critical to ensure homolog pairing and synapsis in early prophase I [20]. Early in mouse meiotic prophase, homologous chromosomes move and cluster through a limited sector of the NE (i.e. Bouquet stage) which is temporally correlated with chromosome alignment and homolog pairing. Pairing of homologous chromosomes is then stabilized by the assembly of the synaptonemal complex (SC) to ensure the process of homologous recombination. Remarkably, in mouse mutants that lack axial element proteins of the SC, synapsis and recombination are disrupted. The attachment of telomeres to the NE is apparently not affected, however [21]. This observation provides additional support to the hypothesis that the TERB1-TERB2-MAJIN complex is responsible for meiotic telomere anchoring to the NE.

The SUN-KASH proteins, are widely conserved in animals, nematodes, yeast, and plants [22–24]. The conservation of the meiotic specific telomere adaptor proteins is less clear, however. In yeast, a similar mechanism for anchoring telomeres to the NE has been identified. It involves the Bqt1–2 meiotic telomere adaptor that connects the Taz1-Rap1 telomere protein to the Sad1 SUN domain protein in a complex with the INM proteins Btq3–4, [25–29] (Fig. 1b-c). No protein sequence similarity between these yeast telomere adaptor proteins and those of mice has been, detected, however.

The high divergence between yeast and mouse telomere adaptor proteins makes it unclear how conserved are the mechanisms mediating the dynamic anchoring of meiotic telomeres to the NE. To probe the origin of the murine TERB1-TERB2-MAJIN complex, we used computational methods to identify their putative orthologs in public databases and subjected candidates to expression studies. Our results indicate that the TERB1-TERB2-MAJIN complex is evolutionarily ancient and that dates back to the common ancestor of metazoans.

## Results

### TERB1, TERB2, and MAJIN are ancestral metazoans proteins

To identify candidate TERB1, TERB2 and MAJIN homologs in other taxa, we carried out a bioinformatic screen of public sequence databases using PSI-BLAST [30]. Our survey of public sequence databases identified homologs of the mouse meiotic telomere complex proteins TERB1, TERB2, and MAJIN mainly in metazoans (Supplementary Information, Table S1, S2 S3, and S4). Most sequences were obtained from Deuterostomes and especially in the Vertebrata, but candidates were also readily identified in Cephalochordata, Echinodermata, and Hemichordata. Beside the Deuterostomes, homologs were also detected in the Lophotrochozoa principally Mollusca, Annelida, and Brachiopoda. Only a few putative homologs were found among Ecdysozoans in the Priapulida and Arthropoda clades. We also identified putative homologs of TERB1, TERB2, and MAJIN in non-bilaterians such as Cnidaria, Placozoa, and Porifera. The taxonomic distributions of TERB1, TERB2, and MAJIN were very similar, meaning that all three corresponding genes are present in metazoan species. Of note, TERB1, TERB2, and MAJIN are present in a single copy in vertebrates, despite the two rounds of whole genome duplication that occurred during the evolutionary history of this group [31]. This indicates that paralogs resulting from these events were not retained during evolution. Similarly, no paralogs were observed in other metazoan lineages.

These results implied that the three proteins are likely ancient in metazoans and arose before the emergence of bilaterians.

**Fig. 2:**
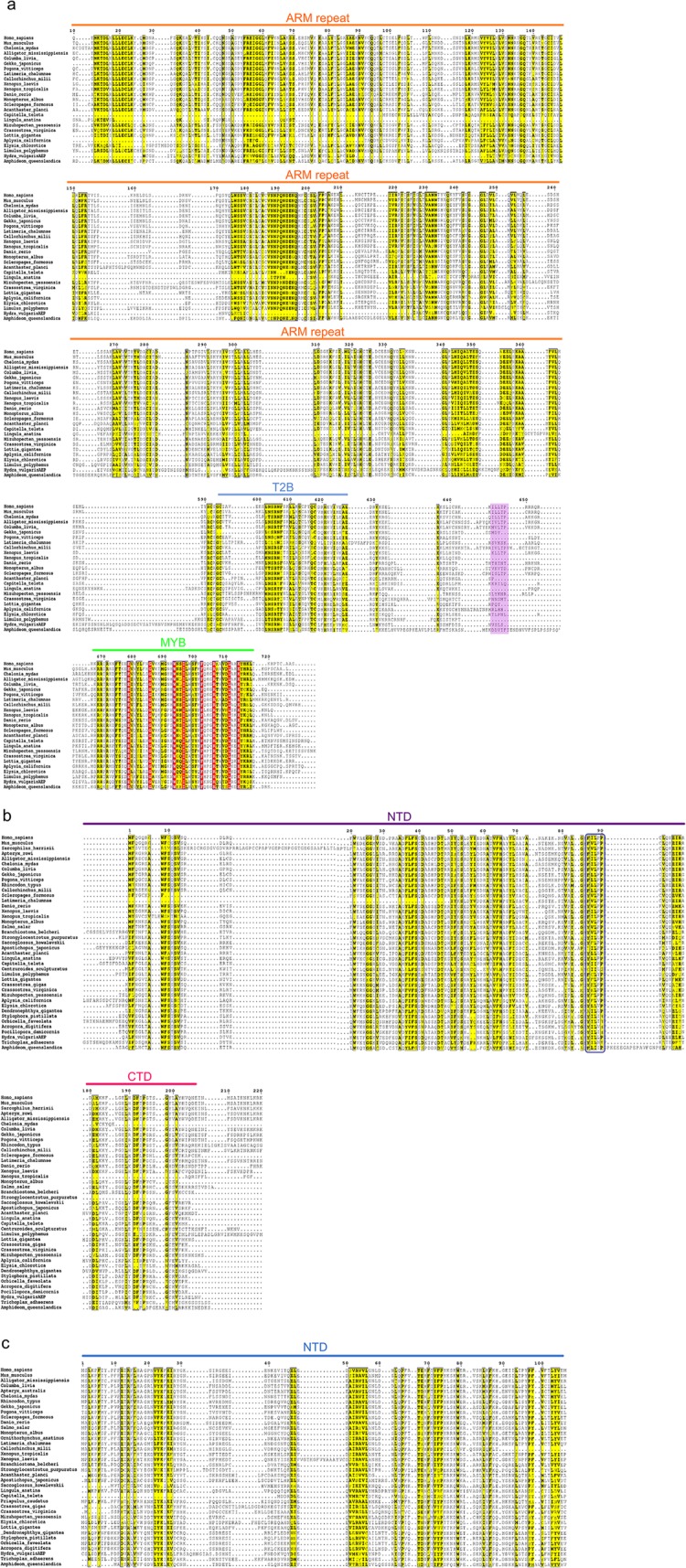
TERB1-TERB2-MAJIN proteins share domain architecture organization and functional sites across metazoans. Multiple sequence alignment of the most conserved regions of putative orthologs of mice TERB1 (**a**), TERB2 (**b**), and MAJIN (**c**) from various representative metazoan species with PROMALS3D and annotated using ESPrit 3.0. The threshold for grouping of the residues was set to 70% and is depicted in yellow. Amino acid positions conserved in all taxa are highlighted in red. Species names are shown at the left. The horizontal lines above the alignment indicate domain boundaries. Armadillo repeat; ARM repeat, T2B; binding site of TERB2 and MYB; homeodomain; N-terminal domain (NTD); and C-terminal domain (CTD). The TRF1-binding motif region of mouse TERB1 is highlighted by the violet rectangle. The motif [F/YxLxP] detected in all TERB2 sequences is shown in the dark blue rectangle

TERB1 multiple sequence alignments revealed high similarity in protein domain organization with N-terminal ARM repeats (aa 16–384) and a C-terminal MYB (aa 715–747) domain shared between Porifera, Cnidaria (Hydrozoans), Annelida, Mollusca, Brachiopoda, Echinodermata (Asterozoa) and Vertebrata (Fig. 2a, Supplementary Fig.S1). Surprisingly, candidate TERB1 sequences from Cnidaria (Anthozoa), Arthropoda, Priapulida, Cephalopoda, Hemichordate and Cephalochordate appear to lack a MYB domain (Supplementary Information, Table S2). Nonetheless, closer inspection of the sequence alignment indicated amino acid conservation flanking the essential site for TERB2 binding (T2B) (aa 593–622 of mice) in TERB1 [14–17] (Supplementary Fig. S2). The probable absence of a MYB domain suggests either annotation errors or the loss of this domain during evolution. In addition, TERB1 interacts directly with the shelterin complex protein TRF1 via its TRFB domain, (aa 523–699 of mice) which is adjacent to the TERB2 binding site (T2B) near its C-terminus [13–17]. Previous biochemical and structural studies have analyzed the interaction between TERB1 and TRF1 using a short peptide sequence (IxLxP, aa 646–650 of human) that highly resembles the TRF1-binding motif [F/YxLxP], of the shelterin-associated protein TIN2 [15, 32, 33]. This motif was conserved mostly in Vertebrata, however (Fig. 2a, Supplementary Fig. S1).

The multiple alignment of TERB2 sequences revealed prominent conservation stretches spanning the entire N-terminal domain (aa 1–116 of mouse) (Fig. 2b). Furthermore, smaller stretches along the C-terminal region of TERB2 (aa 174–209 of mice) were found to be conserved among taxa (Fig. 2b, Supplementary Fig. S3). This result indicates that the most conserved features in TERB2 comes from the protein regions essential for the interaction with TERB1 and with MAJIN [14, 18, 19]. Unexpectedly, at the very end of the N-terminal domain (human and mouse aa 86–90) we noted in all taxa a motif equivalent to [F/YxLxP], involved in the binding of shelterin-associated proteins to the TRFH domain of TRF1 and TRF2 (Fig. 2b) [32]. This finding suggests that telomeric shelterin proteins TRF1 could potentially recruit TERB2 in addition to TERB1 (see above).

Finally, a multiple sequence alignment of MAJIN candidates showed sequence similarity mostly to the N-terminus (aa 2–194 of mice) (Fig. 2c, Supplementary Fig. S4). This region interacts with the C-terminal part of TERB2 [14, 18]. In addition, mouse MAJIN features a single hydrophobic TM-segment on its C-terminus (TM, aa 232–251) [14]. The multiple sequence alignment of MAJIN sequences revealed moderate conservation among taxa in the stretch harboring the TM domain (Supplementary Fig. S4). Secondary structure predictions of the multiple sequence alignment suggested a helix structure in this region which would be consistent with the presence of a potential transmembrane domain (Supplementary Fig. S4). These result suggests that MAJIN’s DNA binding properties and INM anchoring mechanism are conserved among metazoans.

Overall, the multiple sequence alignment of putative TERB1-TERB2-MAJIN orthologs showed comparable protein organization to the mouse proteins and revealed conserved stretches of high similarity in those domains required for the interaction with binding partners. These findings support the notion that despite their great divergence, these proteins are bona fide homologous, meaning that they derive from a common ancestor.

**Fig. 3:**
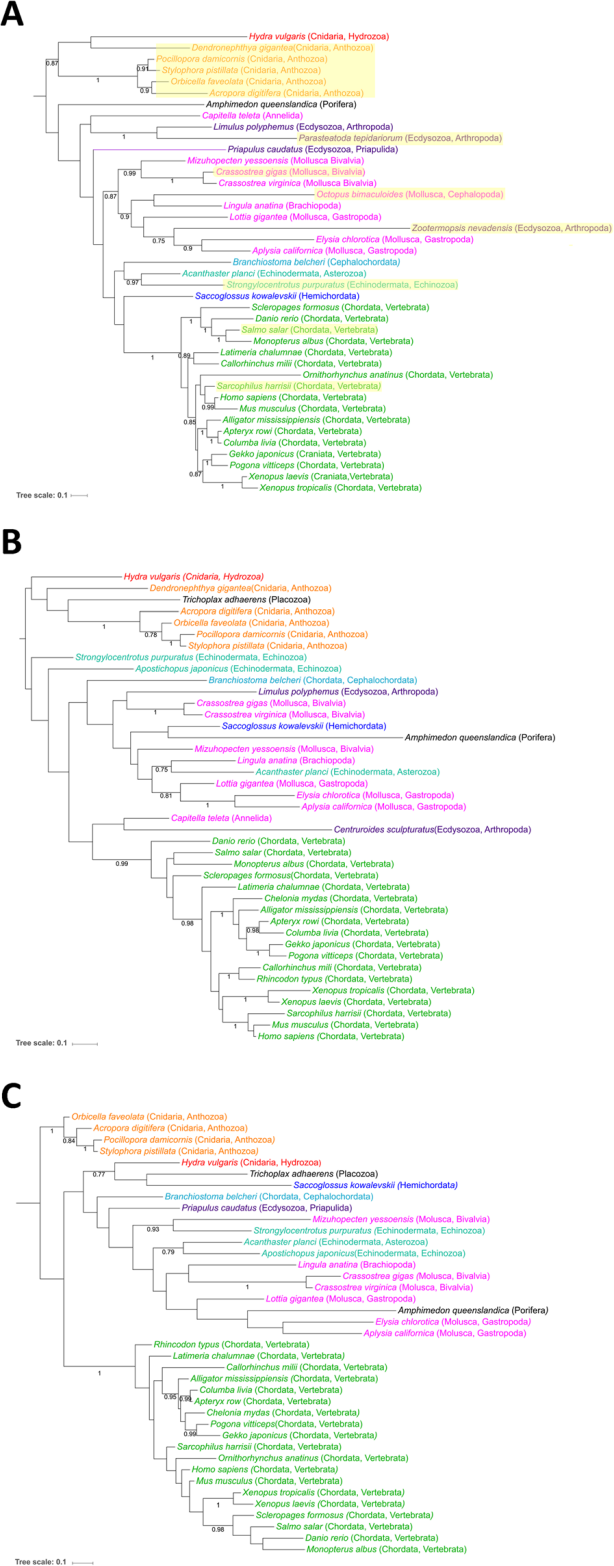
Evolutionary history of metazoan TERB1, TERB2, and MAJIN proteins. Unrooted Bayesian tree of TERB1 (**a**), TERB2 (**b**) and MAJIN (**c**) proteins inferred with MrBayes. Numbers at branches represent posterior probabilities. For clarity, values of < 0.75 were omitted from the tree. The taxa lacking a MYB domain for TERB1 are highlighted in yellow. The length of each branch is proportional to the number of amino acid substitutions per site that have occurred. The bar displays the average number of substitutions per site (scale at botton left of each panel)

To recover the evolutionary history of TERB1, TERB2, and MAJIN proteins, taxonomically balanced Bayesian trees were built (Fig. 3a-c). The deepest nodes of the trees (especially TERB2 and MAJIN) were poorly resolved (weak posterior probabilities < 0.75), probably due to the relatively small number of sites that were kept for the phylogenetic analyses and indicating a lack of phylogenetic signal rather than a true conflicting signal (Fig. 3a-c). Nevertheless, it was noticeable that within Vertebrata, the relationships among sequences were consistent with the systematics, and that most Lophotrochozoa sequences grouped together (Fig. 3a-c). In the Ecdysozoa clade, candidate proteins that belong to Arthropoda (*Zootermopsis nevadensis* and *Centruroides sculpturatus*) for TERB1 and TERB2 are less conserved and showed relatively long branches indicating suggesting a fast rate of evolution (Fig. 3a-b).

In summary, these results clearly indicated that MAJIN, TERB1, and TERB2 were present in the ancestor of all present-day metazoans and are conserved in lineages including the early-diverging Porifera, Placozoa, and Cnidaria.

### Expression of TERB1, TERB2 and MAJIN in the basal metazoan *Hydra*

According to our analysis, we have identified orthologs of TERB1, TERB2, and MAJIN across metazoans. To provide insight into a possible meiotic role of the candidate orthologs in species evolutionarily distant from the mouse, we decided to investigate the expression pattern of putative TERB1, TERB2 and MAJIN orthologues in the basal metazoan *Hydra vulgaris.* Equal amounts of total RNA were isolated from four different body regions: head; body column; testes; foot. TERB1, TERB2, and MAJIN transcripts were detected by RT-PCR using specific primers that spanned exon-exon junctions in the predicted transcript of *Hydra vulgaris* (Supplementary Information, Table S5).

**Fig. 4:**
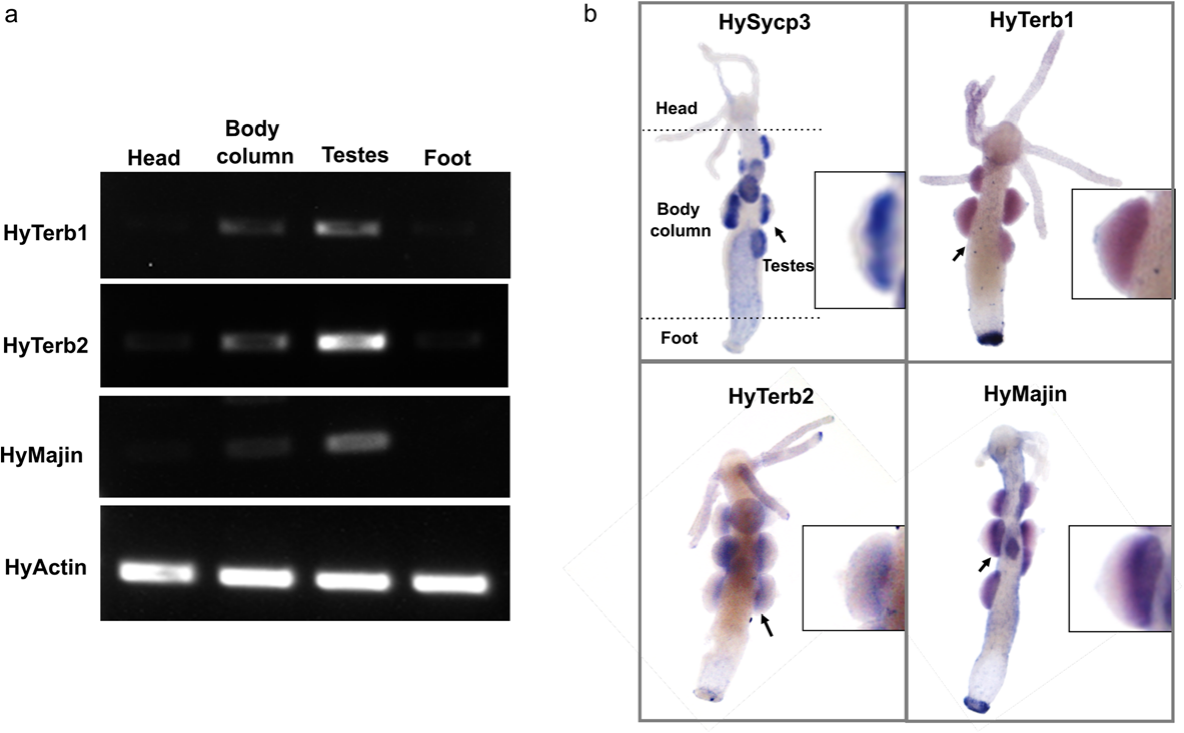
Gonad-specific expression of Terb1, Terb2, and Majin in Hydra vulgaris. **a** Terb1, Terb2, Majin were detected in Hydra tissues by RT-PCR. Actin was used as a control. All three transcripts are exclusively found in the testes. **b** Whole mount in situ hybridization to detect HySycp3, HyTerb1, HyTerb2, and HyMajin transcripts. Arrows indicate the regions shown enlarged in the insets. All four transcripts were detected in cells at the base of the testes

Our experiments showed that putative Hydra Terb1, Terb2 and Majin were specifically detected in the testis fraction. Faint signals in the body column fraction might result from slight contamination with testis tissue. Amplification of Hydra Actin demonstrated the equal concentration of mRNA in the different fractions (Fig. 4a).

To confirm this expression pattern and to evaluate the spatial localization of Hydra Terb1, Terb2, and Majin transcripts we performed whole mount in situ hybridization (WMIH) (Fig. 4b). Sycp3, a specific marker of meiotic cells, was used as a positive control [34]. Transcript signals corresponding to Terb1, Terb2, Majin and Sycp3 were restricted to the basal layer of the testes, i.e. the site where meiotic cells are located [35, 36].

In addition, we managed to amplify the complete coding sequences of *Hydra vulgaris* Terb1, Terb2, and Majin using the predicted ORF sequences (Supplementary Fig. S5) from testis fraction. The predicted translation product from our full-length cDNA experiments showed similar domain organization to the mouse TERB1, TERB2, and MAJIN protein sequences (Supplementary Fig. S6).

Taken together, our results are consistent with the notion that TERB1, TERB2 and MAJIN would fulfill meiosis-specific functions across metazoans.

## Discussion

### TERB1-TERB2 and MAJIN date back to the common ancestor of metazoans

Active chromosome movements during meiotic prophase I is an evolutionarily conserved hallmark that ensures fidelity in the pairing of homologous chromosomes, synapsis, and recombination. Despite the biological conservation of telomere anchoring to the NE, the sequences of meiotic-specific telomere adaptor proteins are highly divergent between mouse and yeast. Our results casts new light on the evolution of the TERB1-TER2-MAJIN complex supporting the hypothesis of an origin that dates back to the common ancestor of metazoans.

In agreement with the commonly accepted view of basal metazoan phylogeny [37] our analysis roughly recovers metazoan phylogeny separating non-bilaterians among Protostomia (Lophotrochozoa and Ecdysozoa) and Deuterostomia taxa despite the deepest nodes (BPP < 0.75) being poorly resolved. In contrast, for the ecdysozoan clade only a few taxa belonging to Priapulida (*Priapulus caudatus*) and Arthropoda (*Zootermopsis nevadensis*, *Parasteatoda tepidariorum*, *Limulus polyphemus, Centruroides sculpturatus*,) were identified as encoding homologous sequences. Relatively long branches were additionally computed indicating a fast-evolutionary rate, especially for Arthropoda. A possible explanation could be that the ancient meiotic telomere complex was lost/replaced or highly diversified in lineages leading to Nematoda and Arthropoda. This would imply that such functions may have been replaced by non-homologous proteins or have diversified to a degree that the homology cannot be detected. Indeed, several line of evidence showed that *C. elegans* and *D. melanogaster* (well-studied meiotic invertebrate model organisms) possess higher rates of divergence and gene loss in comparison to humans and cnidarians, which have greater gene similarity [38, 39].

The mechanism that the nematode *C. elegans* uses to anchor chromosomes to the NE is through a sub-telomeric region of the chromosome, consisting of short repetitive sequences at one end of each chromosome called pairing centers (PC) [40]. A set of four zinc fingers, (ZIM-1/2/3 and HIM-8) associate with the PCs [41, 42] and recognize a (TTGGC) motif, which is closely related to the telomeric repeat in *C. elegans* [40]. The main role of this zinc finger family is to couple chromosome ends to the LINC complex (SUN-1-ZYG-12) [42, 43] but the direct connectors to the LINC complex [22] in *C. elegans* are still unknown. Conversely in *D. melanogaster* chromosomes do not form a bouquet at meiotic prophase and seems unlikely that telomere-led movements mechanism, plays a direct role in pairing, synapsis, and recombination [44].

Overall, the data obtained revealed that the mouse meiosis-specific telomere complex is not an invention of mammalian lineages but instead originated as early as eumetazoans, pointing to a single origin. It was subsequently either secondarily lost or diverged beyond recognition in lineages leading to *C. elegans* and *D. melanogaster*. The existence of very distantly-related sequences of TERB1, TERB2 and MAJIN in ancient non-bilateral clades such as Cnidaria, Placozoa, and Porifera, make it seem likely that these ancestral meiotic telomere complexes emerged even earlier, in the ancestor of Metazoa. Our experiments demonstrated that basal metazoans such as *Hydra vulgaris* expressed putative orthologs of mouse TERB1, TERB2, and MAJIN. These orthologs were expressed at the base of the testis, the same location where spermatocytes reside. The last observation strongly supports a meiotic role for these genes in Cnidaria.

### TERB1, TERB2, and MAJIN share binding sites sequence domains across metazoans

Mouse TERB1, TERB2 and MAJIN proteins cooperate to establish the meiotic telomere complex by interaction with two other protein complexes: telomeric shelterin and the LINC complex [13–15, 17–19]. TERB1 associates with telomeric DNA and binds both the TRF1 shelterin protein and TERB2-MAJIN. Our sequence analysis detected canonical ARM repeat and MYB-like domain organization of candidate TERB1 sequences in diverse phyla such as Chordata (Vertebrata), Arthropoda (Chelicerate), Mollusca (Gastropod and Bivalves), Annelida, Brachiopoda, *Hydra vulgaris* (Hydrozoa, Cnidaria) and *Amphideom queenslandica* (Porifera). Nevertheless, species that belong to Cnidaria (Anthozoa), Priapulida, Hemichordate, Cephalochordate and some Chordata (Vertebrata) possess TERB1 sequences that likely lost the MYB-like domain. The adaptable armadillo repeats structure enables diverse functions [45] and was suggested to interact with SUN1 [13, 14]. However, MYB domain or telobox consensus was specifically characterized to bind double-stranded telomeric DNA [46]. Indeed, the TERB1 MYB domain associates with the meiosis-specific cohesin component SA3 to provide structural integrity to telomeres during prophase rapid movement [14, 17]. However, the possible absence of the MYB domain in certain taxa remains to be verified.

Moreover, TERB1 localized at telomeres through the interaction with TRF1 [13]. The TRF1-TERB1 association in mammals is through a peptide-docking sequence motif in human/mouse TERB1 (aa 645–648), comparable to the strategy adopted by TIN2 [15, 32]. Despite it being demonstrated that mutations in this motif abolished or substantially impaired the binding of TRF1, leading to a decreased fertility in male mice [15], our analysis failed to find this motif outside the clade of Vertebrata. This discrepancy could be attributed to a new adaption in vertebrate lineages to interact with TRF1. Further research should be done to characterize the mechanistic association and/or regulation in relation to the shelterin complex outside the clade of Vertebrata. Conversely, almost all TERB1 sequences analyzed bear the essential region for TERB2 binding (T2B). Recently, it was reported that the human TERB1 C-terminus (aa 590–649) interacts with the N-terminus of TERB2 (aa 2–116) through hydrophobic contacts and electrostatic interactions [18]. In addition, the structure of T2B of TERB1 was resolved in two helices (H1 and H2), matching the predicted alpha helical secondary structure for this region (Supplementary Information, Fig. S1).

Unexpectedly, our multiple sequence analysis identified a highly conserved motif in the N-terminal domain of TERB2 (aa 86–90 of human) in all taxa. This motif resembles the [F/YxLxP] motif [32] required for shelterin proteins to bind the TRHF interface of TRF1 and TRF2, as previously discussed in mammals (Fig. 2b). These findings suggest that TERB2 could intrinsically be recruited to and/or interact with shelterin TRFH surfaces, although further analysis needs to be done to confirm its functionality in the context of meiosis. In addition, the sequence alignment not only highlighted that the N-terminus of TERB2 was the most conserved region but also detected a short stretch of similarity at the C-terminus which nicely corresponds to the regions interacting with MAJIN. Interestingly, a closer inspection of the identified MAJIN putative orthologs showed that the mostly highly conserved region is the N-terminus which associate with TERB2. On the other hand, mouse MAJIN features a hydrophobic helix TM domain on its C-terminus (TM, aa 232–251). Although our multiple sequence analysis recognized some level of conservation the TM domain in other species remains to be identified.

## Conclusion

Altogether, these results suggest that the TERB1-TERB2-MAJIN complex has an ancient origin in metazoans. Its detection in germ-line tissue of the ancient non-bilaterian *Hydra vulgaris* supports functional conservation over evolutionary time, implicating it as a critical mediator of meiotic chromosome attachment.

## Methods

### Data mining

The non-redundant protein database from the NCBI (https://www.ncbi.nlm.nih.gov/) was used to retrieve homologs of mice TERB1 (NP_851289), TERB2 (NP_083190), and MAJIN (NP_001159391) using PSI-BLAST (options: substitution matrix = BLOSUM45, word size = 3) [30]. Iterations were repeated for newly detected homolog sequences until convergence. Genomic sequences available at the NCBI (e.g. genome, est., tsa) and ensembl databases, were checked with the TBLASTN algorithm using the BLOSUM45 matrix. All retrieved sequences were used for reciprocal BLAST tests to ensure that they represented putative homologues of TERB1, TERB2, and MAJIN proteins and not false positives. *Hydra vulgaris* TERB1, TERB2, and MAJIN sequences obtained from this study (see below) were included in the analysis.

### Sequence alignment and phylogenetic tree construction

The retrieved sequences were aligned using PROMALS3D [47] which takes into account data from structural information for better understanding of sequence-structure-function relationships in distant homologues. Available structures from the Protein Data Bank (PDB) [48] for TERB1 (: 1x58_chainA and 6j07_chainB), TERB2 (6j07_chainA) and MAJIN (6j08_chainA) were uploaded in PROMALS3D. Annotations of the sequence alignment were designed using ESPript3 [49]. Protein motifs and functional domains were examined through the Eukaryotic Linear Motif (ELM) (http://elm.eu.org/index.html) [50] and Superfamily web server (http://supfam.org/) [51] respectively.

For phylogenetic analyses, we selected a taxonomically balanced subset of homologous sequences which represent a wide range of animal phyla (Supplementary Information, Table S1, Table S3, and Table S4). The sequences were aligned with MAFFT v7.309 with the accurate option L-INS-I. The resulting multiple alignments were trimmed with BMGE (“Block Mapping and Gathering with Entropy” software) v.1.12 (option -m BLOSUME45) [52]. A Bayesian tree was computed with MrBayes v3.2.6 [53] with a mixed model of amino acid substitution including a gamma distribution (4 discrete categories) MrBayes was run with four chains for 1 million generations and trees were sampled every 100 generations. To construct the consensus tree, the first 2000 trees were discarded as “burn in”. The final trees were drawn with iToL v4 [54].

### Maintenance of *Hydra vulgaris* strain AEP

Experiments were carried out using *Hydra vulgaris* strain AEP [55]. Animals were cultured according to standard procedures at 18 °C [56]. Sexual differentiation was induced by feeding the animals daily for at least 1 week, then starving them for up to 5 days starving the animals [57].

### Isolation of RNA, reverse transcription, PCR, and cloning cDNA

Total RNA from *Hydra vulgaris* strain AEP, head, body column, testes and foot tissue were isolated of approximately 50–80 hydras using a peqGOLD TriFast kit (PeqLab) according to the manufacturer’s protocol. The RNA quality from different fractions was evaluated by non-denaturing agarose gel electrophoresis Subsequently, complementary DNA (cDNA) was synthesized from 1 μg of RNA, Oligo (dT)18 primer (Fermentas) and the M-MLV reverse transcriptase (Promega) according to the manufacturer’s instructions.

The cDNAs obtained were used as templates for the identification of the complete coding sequences and expression analyses in different tissues. To amplify full length coding sequence of *Hydra vulgaris* we designed specific primers using the predicted ORF sequences (Supplementary Information, Table S5). The cDNA amplification was performed with Phusion polymerase (Thermo-Fisher). PCR products were visualized on 1% agarose gel (peqLab) to verify size (Supplementary Information, Fig. S7), purified with a NucleoSpin Gel and PCR Clean-up Kit (Macherey-Nagel) and inserted into the pSC-A-amp/kan plasmid using a StrataClone PCR Cloning Kit (Agilent Technologies). The plasmids were purified using a NucleoSpin® Plasmid kit (Macherey-Nagel) kit and verified by automated sequencing. To verify the obtained full-length or nearly full-length of cDNA, single read sequences from independent cloning were compared with the transcript reference sequence.

The expression studies were performed by RT-PCR using an intron spanning primer set (Supplementary Information, Table S5) designed the genomic annotations of Hydra vulgaris, TERB1 (Genbank ID: NW_004171015), TERB2 (Genbank ID: NW_0041710153) and MAJIN (Genbank ID: NW_004173123). *Hydra* actin was used as a housekeeping gene to control for RNA amounts. The reactions were carried out with Phusion polymerase (Thermo-Fisher) for 32 cycles, so the amplification product was clearly visible. The products were visualized in a 1.2% agarose gel (PeqLab).

### Whole mount in situ hybridization

Antisense probes were generated by cloning a part of the putative *Hydra* homologs of TERB1, TERB2, and MAJIN (size between 400 and 700 bp) into the pSC-A-amp/Kan vector (StrataClone PCR Cloning Kit, Agilent) using specific primers (Supplementary Information, Table S4). After the validation of clone sequence, the vector was linearized for the synthesis of antisense RNA probes. Probes were synthesized by T7 and T3 RNA polymerase (Thermo-Fisher) with the incorporation of Dig-11-UTP (Roche Applied Science) at 37 °C for 2 h. The RNA probes were purified using an RNeasy mini Kit (Qiagen) and checked by non-denaturing agarose gel electrophoresis. In situ hybridizations were performed according to a standard protocol [58] with some modifications. Animals were relaxed in 2% urethane in hydra medium (HM) and fixed overnight with freshly made 4% formaldehyde in HM. The fixed animals were rehydrated and treated with proteinase K (0.05 mg/mL) for 20 min. To halt tissue digestion the animals were incubated with 2 mg/mL glycine solution for 10 min. To minimize the background by preventing binding of the negatively charged RNA probe to positively charged amino groups on proteins samples were incubated in 0.1 M triethanolamine and acetic anhydride (0.25 and 0.5% in 0.1 M triethanolamine) for 10 min each. The samples were re-fixed with 4% formaldehyde for 1 h at room temperature. Before hybridization the samples were washed with hybridization buffer (50% Formamide, 25% 20x SSC, 0.1% Tween 20, 0.15 mg/mL Heparin, 5 mg/mL Torula RNA) at room temperature for 10 min and with pre-heated hybridization buffer at 57 °C for 1 h. Denatured digoxigenin (DIG)-labeled probes were added to the hybridization solution at a final concentration of 100 ng/mL, and incubated for 20 h at 57 °C. After several washes the samples were blocked in 1% blocking reagent in Maleic acid buffer (MAB: 100 mM Maleic acid, 150 mM NaCl, pH 7.5) for 1 h at room temperature. For the detection of the DIG-labeled RNA probes an anti-DIG antibody coupled to alkaline phosphatase was preabsorbed 1:600 in (1X PBS, pH 7.5) on fixed animals overnight at 4 °C and used directly in the same concentration at 4 °C overnight. Unbound antibody was removed by washing 15 times each for 20 min in 1X PBS, 0.1% (v/v) Triton X-100. Detection of the signal was achieved through first equilibrating the animals with NTM (0.1 M NaCl, 0.1 M Tris-HCl, pH 9.5) for 10 min at room temperature and then incubated in 2% NBT/BCIP (Roche) in the dark. After reaching the optimal signal-to-background ratio, the reaction was stopped by washing the animals in ddH2O, followed by rehydration and mounting on microscope slides in 90% glycerol/1XPBS. The digital images were acquired using a Leica EC3 digital camera mounted on an Olympus SZ61 Zoom Stereo Microscope.

## Supplementary information

**Additional file 1.**

## Data Availability

The datasets generated during and/or analyzed during the current study are included in the supplementary information file. *Hydra vulgaris* MAJIN (Seq1 ID: 2327888), TERB1 (Seq2 ID: 2328111), and TERB2 (Seq3 ID: 2328135) were submitted to GenBank database.

## Abbreviations

ARM: Armadillo repeats
BPP: Bayeasian posterior probabilities
cDNA: Complementary DNA
CTD: C-terminal domain
DIG: Digoxigenin
EtBr: Ethidium bromide
INM: Inner nuclear membrane
LINC: Linker of Nucleoskeleton and Cytoskeleton
MAJIN: Membrane anchored junction protein
MYB: Homeobox-like domain
NE: Nuclear envelope
NTD: N-terminal domain
ONM: Outer nuclear membrane
ORF: Open reading frame
T2B: Site of TERB2 binding in TERB1
TERB1: Telomere repeat binding bouquet formation protein 1
TERB2: Telomere repeat binding bouquet formation protein 2
TRFH: Telomeric repeat factors homology domain
TM: Transmembrane domain
RT-PCR: Reverse transcription polymerase chain reaction
WMIH: Whole mount in situ hybridization
