# Supplementary material for: The TERB1-TERB2-MAJIN complex of mouse meiotic telomeres dates back to the common ancestor of metazoans
Source: BMC Evol Biol. 2020 May 14;20:55. doi: 10.1186/s12862-020-01612-9 (PMC7227075; doi:10.1186/s12862-020-01612-9)
Supplement: Supplementary file 1 — Additional file 1. [file 12862_2020_1612_MOESM1_ESM.docx]

**Supplementary Information**

**The TERB1-TERB2-MAJIN complex of mouse meiotic telomeres dates back to the common ancestor of metazoans**

**Irene da Cruz^1^, Céline Brochier-Armanet^2^ and Ricardo Benavente^1, *^**

^1^ Department of Cell and Developmental Biology, Biocenter, University of Würzburg, 97074 Würzburg, Germany

^2^ Laboratoire de Biométrie et Biologie Evolutive, UMR 5558, Université Lyon 1, F-69622, Villeurbanne, France

**Table S1** Species, taxonomic rank, and protein accession numbers of candidate TERB1 homologues with ARM and MYB domains in metazoans obtained using PSI-BLAST.

| **Species name** | **Taxonomic rank** | **Accesion number** |
| --- | --- | --- |
| *Mus musculus* | Metazoa; Deusterostoma; Chordata; Vertebrata; Mammalia | NP_851289 |
| *Cavia porcellus* | Metazoa; Deusterostoma; Chordata; Vertebrata; Mammalia | XP_003471985 |
| *Homo sapiens* | Metazoa; Deusterostoma; Chordata; Vertebrata; Mammalia | NP_001129977 |
| *Chrysemys picta bellii* | Metazoa; Deuterostoma; Chordata Vertebrata; Testudines | XP_008173404 |
| *Monopterus albus* | Metazoa; Deuterostoma; Chordata; Vertebrata; Actinopterygii | XP_020468880 |
| *Pogona vitticeps* | Metazoa; Deuterostoma; Chordata; Vertebrata; Lepidosauria | XP_020670034 |
| *Scleropages formosus* | Metazoa; Deuterostoma; Chordata; Vertebrata; Actinopterygii | XP_018607988 |
| *Alligator mississippiens* | Metazoa; Deuterostoma; Chordata; Vertebrata; Archosauria | XP_019348499 |
| *Columba livia* | Metazoa; Deuterostoma; Chordata; Vertebrata; Archosauria | XP_021137726 |
| *Callorhincus milii* | Metazoa; Deuterostoma; Chordata; Vertebrata; Chondrichthyes | XP_007906058 |
| *Latimeria chalumnae* | Metazoa; Deuterostoma; Chordata; Vertebrata; Coelacanthimorpha | XP_01434872 |
| *Gekko japonicus* | Metazoa; Deuterostoma; Chordata; Vertebrata; Lepidosauria | XP_015279750 |
| *Chelonya mydas* | Metazoa; Deuterostoma; Chordata; Vertebrata; Testudines | XP_007058728 |
| *Danio rerio* | Metazoa; Deuterostoma; Chordata;Vertebrata; Actinopterygii | NP_001082851 |
| *Xenopus tropicalis* | Metazoa; Deuterostoma; Chordata;Vertebrata; Amphibia | XP_017948679 |
| *Xenopus laevis* | Metazoa; Deuterostoma; Chordata;Vertebrata; Amphibia | OCT57393 |
| *Acanthaster planci* | Metazoa; Eumetazoa; Bilateria; Deuterostomia; Echinodermata | XP_022081545 |
| *Limulus polyphemus* | Metazoa; Eumetazoa; Bilateria; Protostomia; Ecdysozoa; Panarthropoda; Arthropoda | XP_022246046 |
| *Capitella teleta* | Metazoa; Eumetazoa; Bilateria; Protostomia; Lophotrochozoa; Annelida | ELU13718 |
| *Lingula anatina* | Metazoa; Eumetazoa; Bilateria; Protostomia; Lophotrochozoa; Brachiopoda | XP_013407283 |
| *Crassostrea virginica* | Metazoa; Eumetazoa; Bilateria; Protostomia; Lophotrochozoa; Mollusca; Bivalvia | XP_022286282 |
| *Mizuhopecten yessoensis* | Metazoa; Eumetazoa; Bilateria; Protostomia; Lophotrochozoa; Mollusca; Bivalvia | OWF54743 |
| *Aplysia californica* | Metazoa; Eumetazoa; Bilateria; Protostomia; Lophotrochozoa; Mollusca; Gastropoda | XP_005109353 |
| *Lottia gigantea* | Metazoa; Eumetazoa; Bilateria; Protostomia; Lophotrochozoa; Mollusca; Gastropoda | XP_009053858 |
| *Elysia chlorotica* | Metazoa; Eumetazoa; Bilateria; Protostomia; Lophotrochozoa; Mollusca; Gastropoda | RUS80343/EGW08_011882 |
| *Hydra vulgaris* | Metazoa; Eumetazoa; Cnidaria; Hydrozoa | XP_012561908 |
| *Amphimedon queenslandica* | Metazoa; Porifera; Demospongiae | XP_019850131 |
| *Sarcophilus harrisii* | Metazoa; Deuterostoma; Chordata; Vertebrata; Mammalia | XP_012395745 |
| *Ornithorhynchus anatinus* | Metazoa; Deuterostoma; Chordata; Vertebrata Mammalia; Monotremata | XP_007665674 |
| *Salmo salar* | Metazoa; Deuterostoma; Chordata; Vertebrata; Actinopterygii | XP_014004017 |
| *Apteryx australis mantelli* | Metazoa; Deuterostoma; Chordata; Vertebrata; Archosauria | XP_013796592 |
| *Rhincodon typus* | Metazoa; Deuterostoma; Chordata; Vertebrata; Chondrichthyes | XP_020383869 |
| *Branchiostoma belcheri* | Metazoa; Deuterostoma; Chordata; Cephalochordata | XP_019613458 |
| *Branchiostoma floridae* | Metazoa; Deuterostoma; Chordata; Cephalochordata | XP_002588932 |
| *Strongylocentrotus purpuratus* | Metazoa; Eumetazoa; Bilateria; Deuterostomia; Echinodermata | XP_003731208 |

**Table S2** Species, taxonomic rank, and protein accession numbers of candidate TERB1 homologues with ARM domain in metazoans obtained using PSI-BLAST.

| *Saccoglossus kowalevskii* | Metazoa; Eumetazoa; Bilateria; Deuterostomia; Hemichordata | XP_006825363 |
| --- | --- | --- |
| *Priapulus caudatus* | Metazoa; Eumetazoa; Bilateria; Protostomia; Ecdysozoa | XP_014672679 |
| *Crassostrea gigas* | Metazoa; Eumetazoa; Bilateria; Protostomia; Lophotrochozoa; Mollusca; Bivalvia | XP_019919025 |
| *Zootermopsis nevadensis* | Metazoa; Eumetazoa; Bilateria; Protostomia; Ecdysozoa; Arthropoda; Hexapoda; Insecta | XP_021926856 |
| *Parasteatoda tepidariorum* | Metazoa; Eumetazoa; Bilateria; Protostomia; Ecdysozoa; Arthropoda; Chelicerata; Arachnida | XP_015915639 |
| *Octopus bimaculoides* | Metazoa; Eumetazoa; Bilateria; Protostomia; Lophotrochozoa; Mollusca; Cephalopada | KOF89697 |
| *Dendronephthya gigantea* | Metazoa; Eumetazoa; Cnidaria; Anthozoa | XP_028414065 |
| *Acropora digitifera* | Metazoa; Eumetazoa; Cnidaria; Anthozoa | XP_015752571 |
| *Exaiptasia pallida* | Metazoa; Eumetazoa; Cnidaria; Anthozoa | KXJ09956 |
| *Nematostella vectensis* | Metazoa; Eumetazoa; Cnidaria; Anthozoa | XP_001626728 |
| *Orbicella faveolata* | Metazoa; Eumetazoa; Cnidaria; Anthozoa | XP_020616593 |
| *Stylophora pistillata* | Metazoa; Eumetazoa; Cnidaria; Anthozoa | XP_022802203 |
| *Pocillopora damicornis* | Metazoa; Eumetazoa; Cnidaria; Anthozoa | XP_027049808 |
| *Trichoplax adherens* | Metazoa; Placozoa; Trichoplax | XP_002117303 |

**Table S3** Species, taxonomic rank, and protein accession numbers of candidate TERB2 homologues in metazoans obtained using PSI-BLAST

| **Species name** | **Taxonomy rank** | **Accesion number** |
| --- | --- | --- |
| *Mus Musculus* | Metazoa; Deuterostoma; Chordata; Vertebrata; Mammalia | NP_083190 |
| *Branchiostoma belcheri* | Metazoa; Deuterostoma; Chordata; Cephalochordata | XP_019615255 |
| *Monopterus albus* | Metazoa; Chordata;Craniata; Vertebrata; Actinopterygii | XP_020462382 |
| *Latimeria chalumnae* | Metazoa; Deuterostoma; Chordata; Vertebrata; Coelacanthimorpha | XP_006013910 |
| *Gekko japanicus* | Metazoa; Deuterostoma; Chordata; Vertebrata; Lepidosauria | XP_01526237 |
| *Pogona vitticeps* | Metazoa; Deuterostoma; Chordata; Vertebrata; Lepidosauria | XP_020663749 |
| *Scleropages formosus* | Metazoa; Deuterostoma; Chordata; Vertebrata; Actinopterygii | XP_018595098 |
| *Salmo salar* | Metazoa; Deuterostoma; Chordata; Vertebrata; Actinopterygii | XP_014030940 |
| *Alligator mississippiensis* | Metazoa; Deuterostoma; Chordata; Vertebrata; Archosauria | XP_014455423 |
| *Apteryx rowi* | Metazoa; Deuterostoma; Chordata; Vertebrata; Archosauria | XP_025914060 |
| *Apteryx austalis mantelli* | Metazoa; Deuterostoma; Chordata; Vertebrata; Archosauria | XP_013811644 |
| *Columba livia* | Metazoa; Deuterostoma; Chordata; Vertebrata; Archosauria | XP_021139078 |
| *Callorhinchus millii* | Metazoa; Deuterostoma; Chordata; Vertebrata; Chondrichthyes | XP_007906225 |
| *Bos taurus* | Metazoa; Deuterostoma; Chordata; Vertebrata; Mammalia | NP_001070546 |
| *Capra hircus* | Metazoa; Deuterostoma; Chordata; Vertebrata; Mammalia | XP_005686283 |
| *Cavia porcellus* | Metazoa; Deuterostoma; Chordata; Vertebrata; Mammalia | XP_003471804 |
| *Homo sapiens* | Metazoa; Deuterostoma; Chordata; Vertebrata; Mammalia | NP_689661 |
| *Sarcophilus harrisii* | Metazoa; Deuterostoma; Chordata; Vertebrata; Mammalia | XP_012401909 |
| *Chelonya mydas* | Metazoa; Deuterostoma; Chordata; Vertebrata; Testudines | XP_007056532 |
| *Xenopus laevis* | Metazoa; Deuterostoma; Chordata;Vertebrata; Amphibia | XP_018110167 |
| *Xenopus tropicalis* | Metazoa; Deuterostoma; Chordata;Vertebrata; Amphibia | XP_017947896 |
| *Rhincodon typus* | Metazoa; Deuterostoma; Vertebrata; Chondrichthyes | XP_020378706 |
| *Chrysemys picta bellii* | Metazoa; Deuterstoma; Chordata; Vertebrata; Testudines | XP_008165062 |
| *Acanthaster planci* | Metazoa; Eumetazoa; Bilateria; Deuterostomia; Echinodermata | XP_022105193 |
| *Apostichopus japonicus* | Metazoa; Eumetazoa; Bilateria; Deuterostomia; Echinodermata | PIK5153 |
| *Strongylocentrotus purpuratus* | Metazoa; Eumetazoa; Bilateria; Deuterostomia; Echinodermata | XP_011664353 |
| *Saccoglossus kowalevskii* | Metazoa; Eumetazoa; Bilateria; Deuterostomia; Hemichordata | XP_002730738 |
| *Centruroides sculpturatus* | Metazoa; Eumetazoa; Bilateria; Protostomia; Ecdysozoa; Panarthropoda; Arthropoda | XP_023233003 |
| *Limulus polyphemus* | Metazoa; Eumetazoa; Bilateria; Protostomia; Ecdysozoa; Panarthropoda; Arthropoda | XP_022250037 |
| *Capitella teleta* | Metazoa; Eumetazoa; Bilateria; Protostomia; Lophotrochozoa; Annelida | ELT95246 |
| *Crassostrea gigas* | Metazoa; Eumetazoa; Bilateria; Protostomia; Lophotrochozoa; Mollusca; Bivalvia | XP_019930121 |
| *Crassostrea virginica* | Metazoa; Eumetazoa; Bilateria; Protostomia; Lophotrochozoa; Mollusca; Bivalvia | XP_022329935 |
| *Mizuhopecten yessoensis* | Metazoa; Eumetazoa; Bilateria; Protostomia; Lophotrochozoa; Mollusca; Bivalvia | XP_021360479 |
| *Aplysia californica* | Metazoa; Eumetazoa; Bilateria; Protostomia; Lophotrochozoa; Mollusca; Gastropoda | XP_005090461 |
| *Lottia gigantea* | Metazoa; Eumetazoa; Bilateria; Protostomia; Lophotrochozoa; Mollusca; Gastropoda | XP_009048665 |
| *Elysia chlorotica* | Metazoa; Eumetazoa; Bilateria; Protostomia; Lophotrochozoa; Mollusca; Gastropoda | RUS7892277EGW08_013300 |
| *Acropora digitifera* | Metazoa; Eumetazoa; Cnidaria; Anthozoa | XP_015753906 |
| *Orbicella faveolata* | Metazoa; Eumetazoa; Cnidaria; Anthozoa | XP_020630061 |
| *Dendronephthya gigantea* | Metazoa; Eumetazoa; Cnidaria; Anthozoa | XP_028398919 |
| *Pocillopora damicornis* | Metazoa; Eumetazoa; Cnidaria; Anthozoa | XP_027045077 |
| *Stylophora pistillata* | Metazoa; Eumetazoa; Cnidaria; Anthozoa | XP_022784010 |
| *Hydra vulgaris* | Metazoa; Eumetazoa; Cnidaria; Hydrozoa | XP_002169769 |
| *Lingula anatina* | Metazoa; Lophotrochozoa; Brachiopoda | XP_013380855 |
| *Trichoplax adherens* | Metazoa; Placozoa; Trichoplax | XP_002110273 |
| *Amphimedon queenslandica* | Metazoa; Porifera; Demospongiae | XP_019856173 |

**Table S4** Species, taxonomic rank, and protein accession numbers of candidate MAJIN homologues in metazoans obtained using PSI-BLAST.

| **Species name** | **Taxonomic rank** | **Accesion number** |
| --- | --- | --- |
| *Mus musculus* | Metazoa; Deuterostoma; Chordata; Vertebrata; Mammalia | NP_001159391 |
| *Callorhinchus milii* | Metazoa; Deuterostoma; Chordata; Vertebrata; Chondrichthyes | XP_007883186 |
| *Chelonia mydas* | Metazoa; Deuterostoma; Chordata; Vertebrata; Testudines | XP_007052730 |
| *Gekko japonicus* | Metazoa; Chordata; Craniata; Vertebrata; Reptilia | XP_015282829 |
| *Alligator mississippiensis* | Metazoa; Deuterostoma; Chordata; Vertebrata; Archosauria | KYO31280 |
| *Pogona vitticeps* | Metazoa; Deuterostoma; Chordata; Vertebrata; Lepidosauria | XP_020652836 |
| *Branchiostoma belcheri* | Metazoa; Deuterostoma; Chordata; Cephalochordata | XP_019625986 |
| *Branchiostoma floridae* | Metazoa; Deuterostoma; Chordata; Cephalochordata | XP_002606604 |
| *Rhincodon typus* | Metazoa; Deuterostoma; Vertebrata; Chondrichthyes | XP_020370007 |
| *Danio rerio* | Metazoa; Deuterostoma; Chordata;Vertebrata; Actinopterygii | XP_017212702 |
| *Monopterus albus* | Metazoa; Deuterostoma; Chordata; Vertebrata; Actinopterygii | XP_020465821 |
| *Salmo salar* | Metazoa; Deuterostoma; Chordata; Vertebrata; Actinopterygii | XP_014012104 |
| *Latimeria chalumnae* | Metazoa; Deuterostoma; Chordata; Vertebrata; Coelacanthimorpha | XP_014349143 |
| *Xenopus leavis* | Metazoa; Deuterostoma; Chordata; Vertebrata; Amphibia | OCT81751 |
| *Xenopus tropicalis* | Metazoa; Deuterostoma; Chordata; Vertebrata; Amphibia | OCA37246 |
| *Apteryx australis mantelli* | Metazoa; Deuterostoma; Chordata; Vertebrata; Archosauria | XP_013799140 |
| *Columba livia* | Metazoa; Deuterostoma; Chordata; Vertebrata; Archosauria | XP_013224740 |
| *Scleropages formosus* | Metazoa; Deuterostoma; Chordata; Vertebrata; Archosauria | XP_018592548 |
| *Bos taurus* | Metazoa; Deuterostoma; Chordata; Vertebrata; Mammalia | XP_015316798 |
| *Capra hircus* | Metazoa; Deuterostoma; Chordata; Vertebrata; Mammalia | XP_017899084 |
| *Cavia porcellus* | Metazoa; Deuterostoma; Chordata; Vertebrata; Mammalia | XP_013006196 |
| *Homo sapiens* | Metazoa; Deuterostoma; Chordata; Vertebrata; Mammalia | XP_005273975 |
| *Saimiri boliviensis* | Metazoa; Deuterostoma; Chordata; Vertebrata; Mammalia | XP_010346585 |
| *Ornithorhynchus anatinus* | Metazoa; Deuterostoma; Chordata; Vertebrata; Mammalia; Monotrema | XP_007662640 |
| *Sarcophilus harrisii* | Metazoa; Deuterostoma; Chordata; Vertebrata; Mammalia | XP_012408262 |
| *Acanthaster planci* | Metazoa; Eumetazoa; Bilateria; Deuterostomia; Echinodermata | XP_022090649 |
| *Apostichopus japonicus* | Metazoa; Eumetazoa; Bilateria; Deuterostomia; Echinodermata | PIK39444 |
| *Stronglocentrotus purpuratus* | Metazoa; Eumetazoa; Bilateria; Deuterostomia; Echinodermata | XP_011668546 |
| *Saccoglossus kowalevskii* | Metazoa; Eumetazoa; Bilateria; Deuterostomia; Hemichordata | XP_006812193 |
| *Priapulus caudatus* | Metazoa; Eumetazoa; Bilateria; Protostomia; Ecdysozoa | XP_014676280 |
| *Capitella teletea* | Metazoa; Eumetazoa; Bilateria; Protostomia; Lophotrochozoa; Annelida | ELT98474.1 |
| *Cassostrea gigas* | Metazoa; Eumetazoa; Bilateria; Protostomia; Lophotrochozoa; Mollusca; Bivalvia | XP_011454168 |
| *Cassostrea virginica* | Metazoa; Eumetazoa; Bilateria; Protostomia; Lophotrochozoa; Mollusca; Bivalvia | XP_022338123 |
| *Mizuhopecten yessoensis* | Metazoa; Eumetazoa; Bilateria; Protostomia; Lophotrochozoa; Mollusca; Bivalvia | XP_021345218 |
| *Aplysia californica* | Metazoa; Eumetazoa; Bilateria; Protostomia; Lophotrochozoa; Mollusca; Gastropoda | XP_012946253 |
| *Elysia chlorotica* | Metazoa; Eumetazoa; Bilateria; Protostomia; Lophotrochozoa; Mollusca; Gastropoda | RUS71040 /EGW08_021198 |
| *Lottia gigantea* | Metazoa; Eumetazoa; Bilateria; Protostomia; Lophotrochozoa; Mollusca; Gastropoda | XP_009058794 |
| *Acropora digitifera* | Metazoa; Eumetazoa; Cnidaria; Anthozoa | XP_015763651 |
| *Orbicella faveolata* | Metazoa; Eumetazoa; Cnidaria; Anthozoa | XP_020615173 |
| *Dendronephthya gigantea* | Metazoa; Eumetazoa; Cnidaria; Anthozoa | RMX50144 |
| *Stylophora pistillata* | Metazoa; Eumetazoa; Cnidaria; Anthozoa | XP_022790937 |
| *Hydra vulgaris* | Metazoa; Eumetazoa; Cnidaria; Hydrozoa | XP_012563853 |
| *Lingula anatina* | Metazoa; Lophotrochozoa; Brachiopoda | XP_013401921 |
| *Trichoplax adherens* | Metazoa; Placozoa; Trichoplax | XP_002109914 |
| *Amphimedon queenslandica* | Metazoa; Porifera; Demospongiae | XP_019850558 |

| **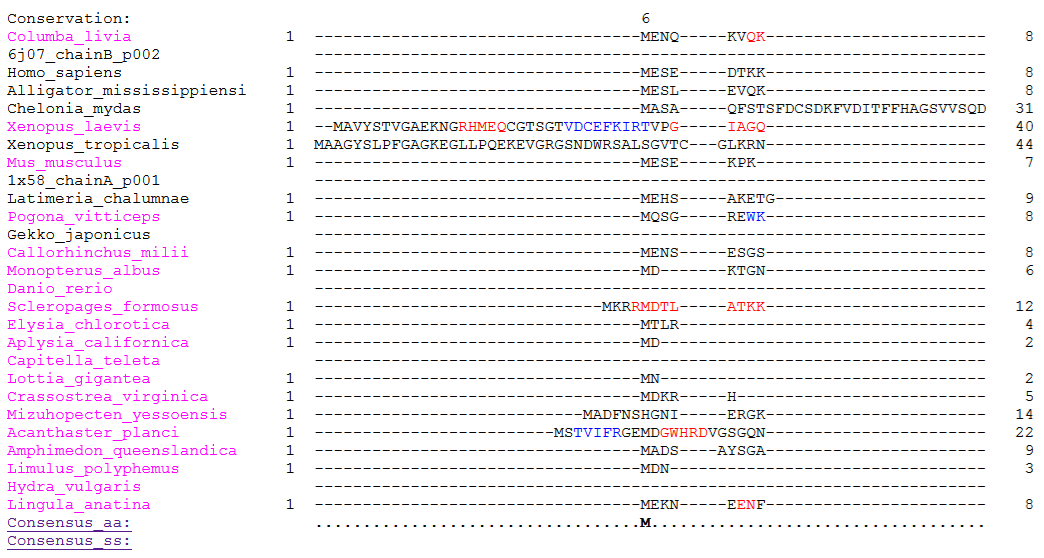** |
| --- |
| **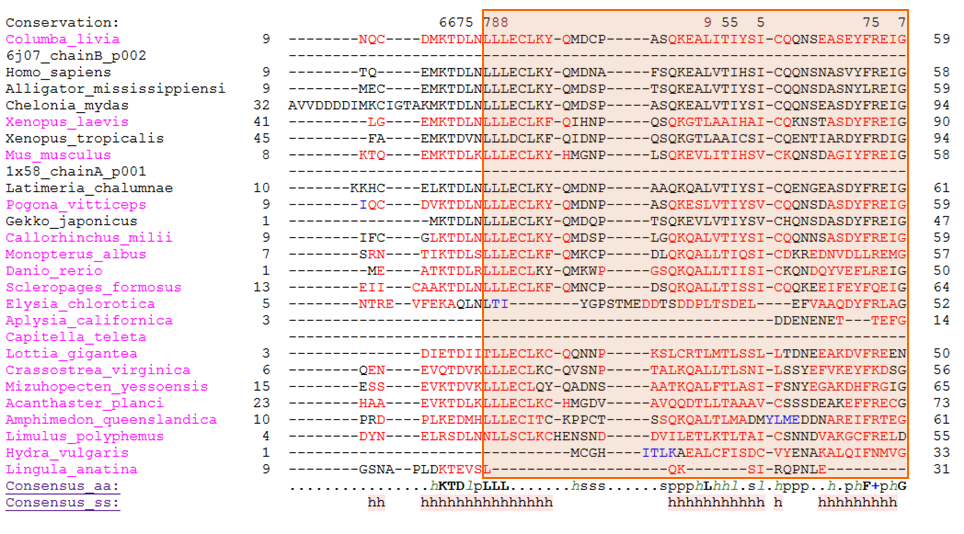** |
| **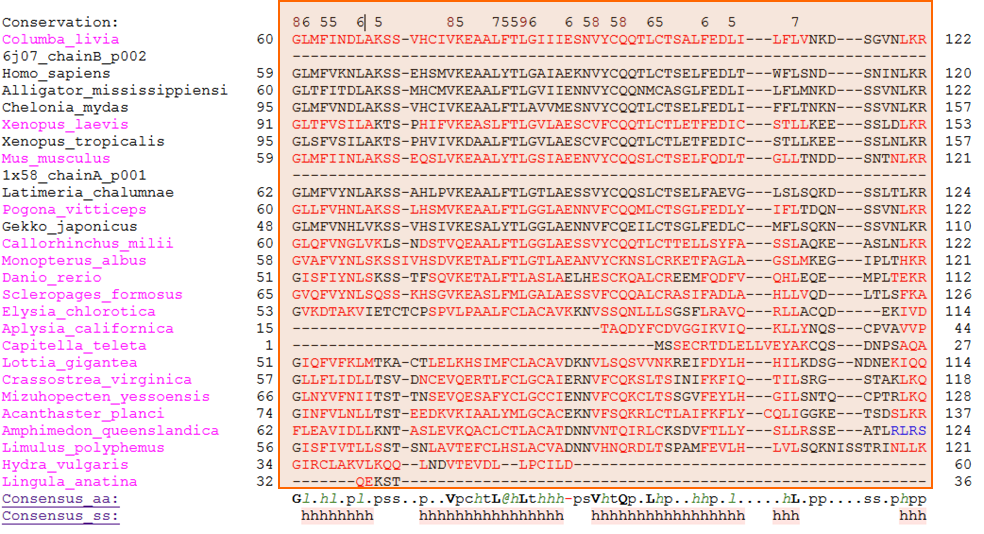** |
| **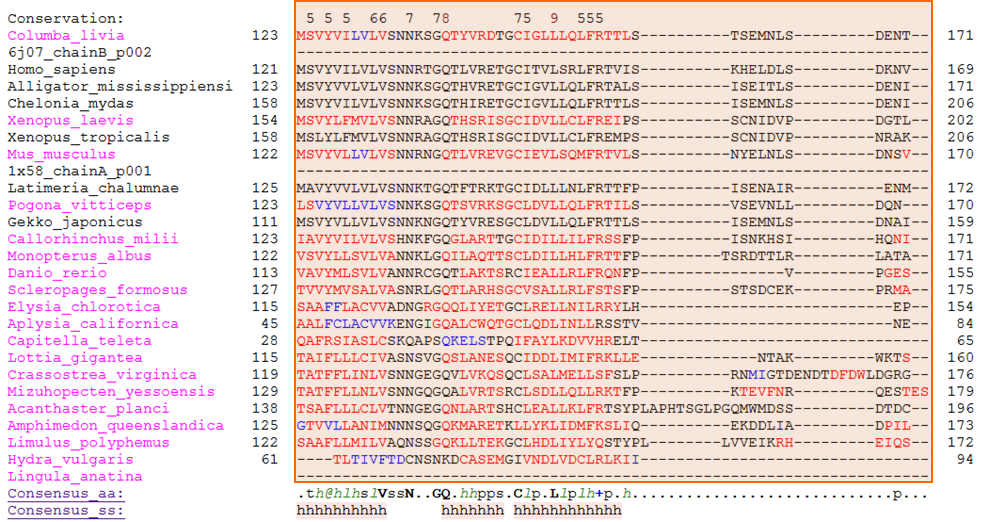** |
| **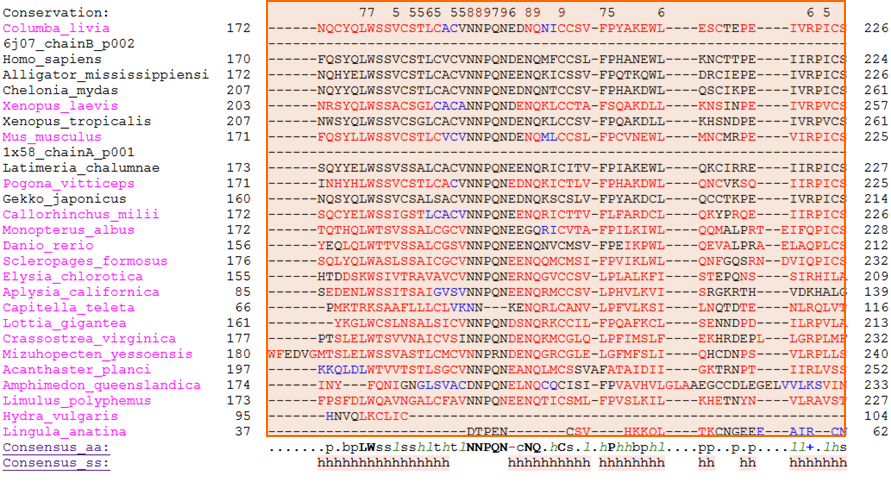** |
| **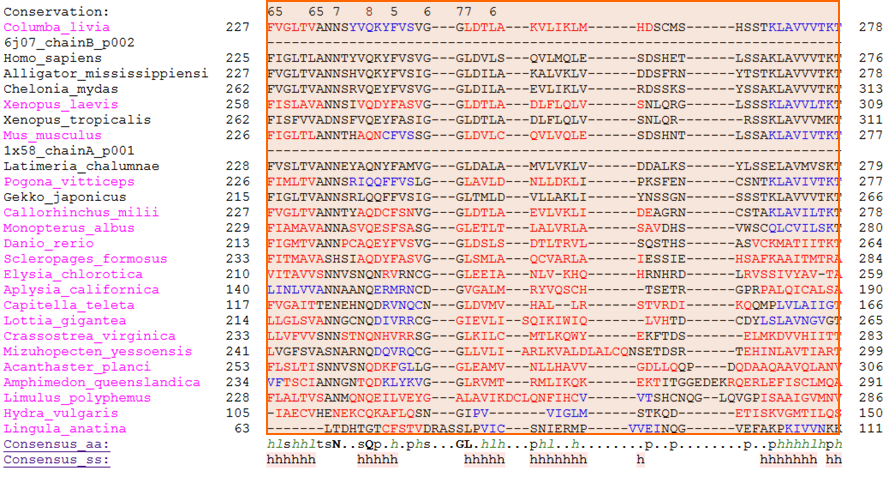** |
| **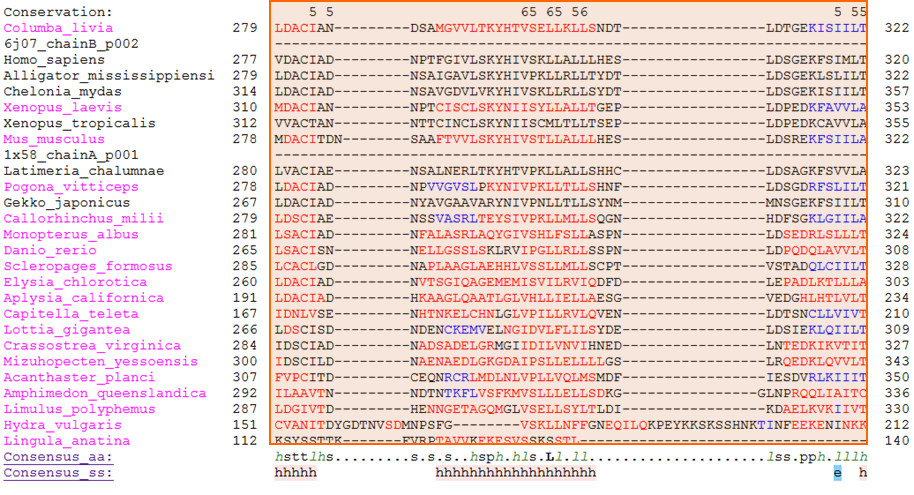** |
| **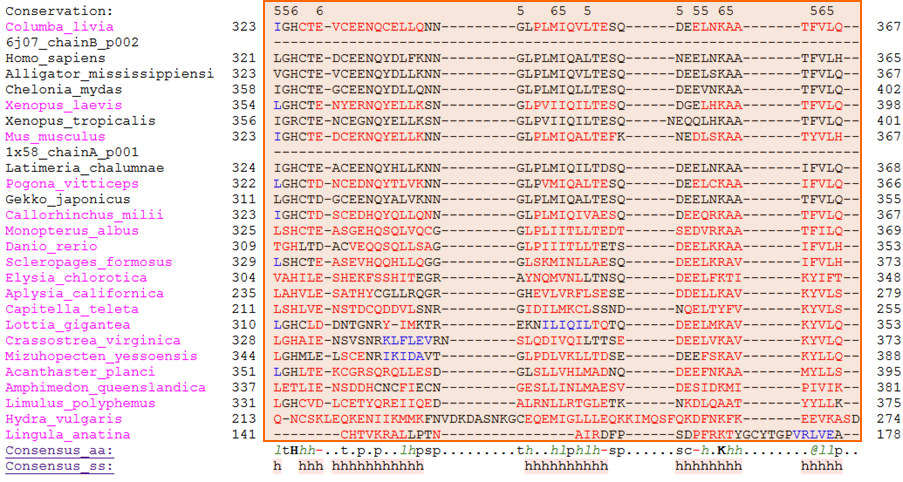** |
| **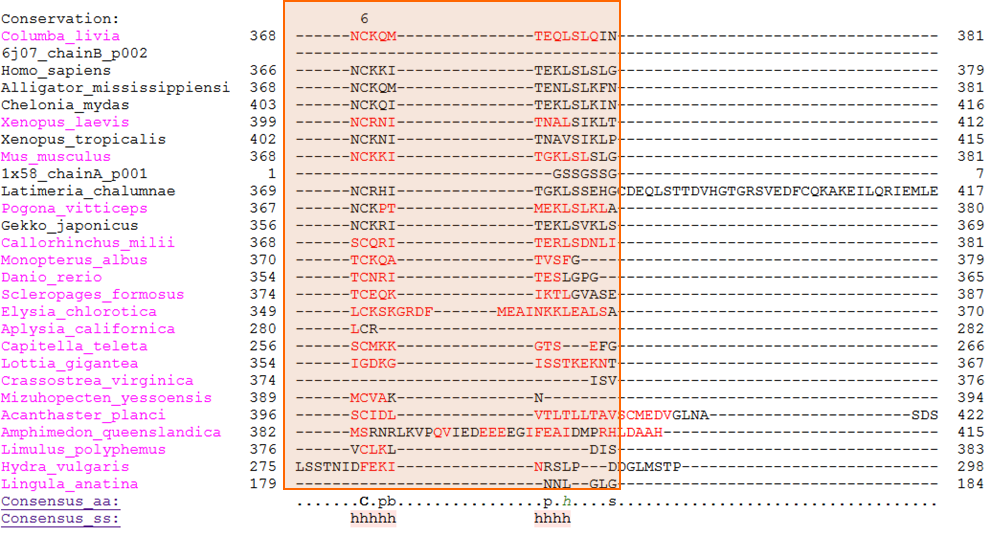** |
| **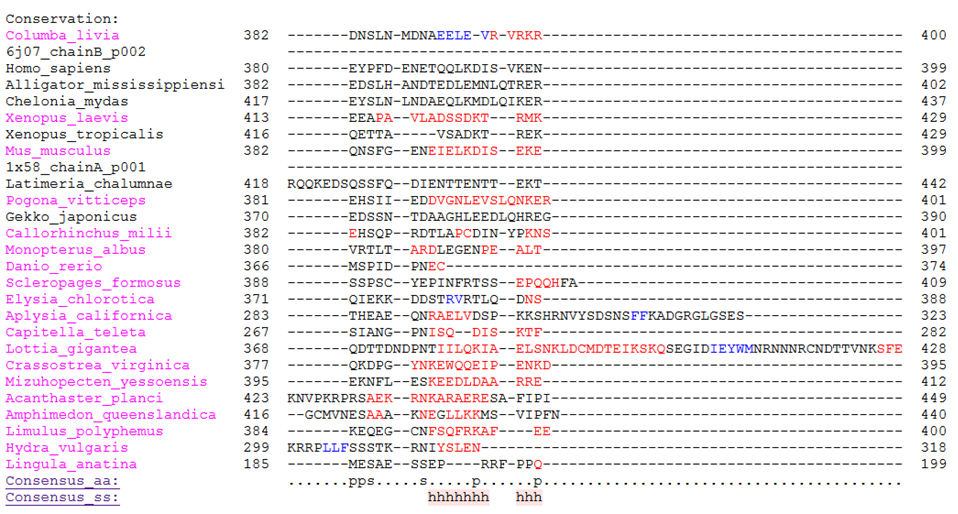** |
| **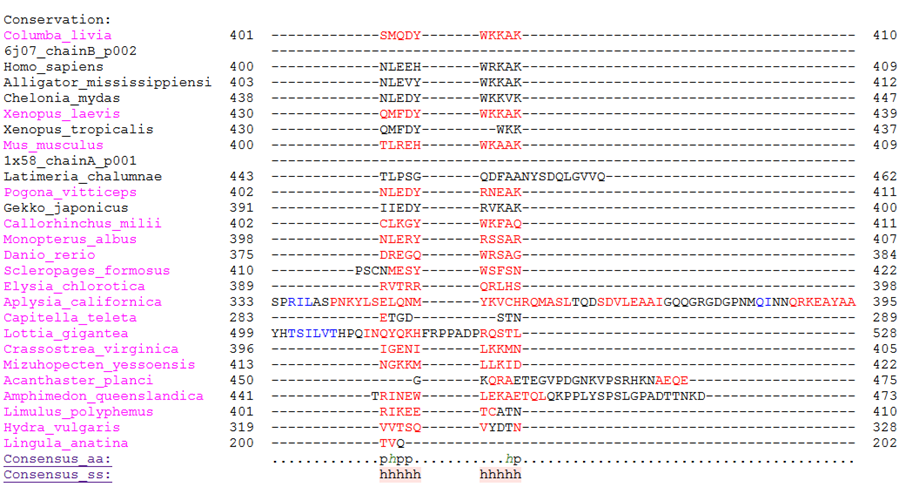** |
| **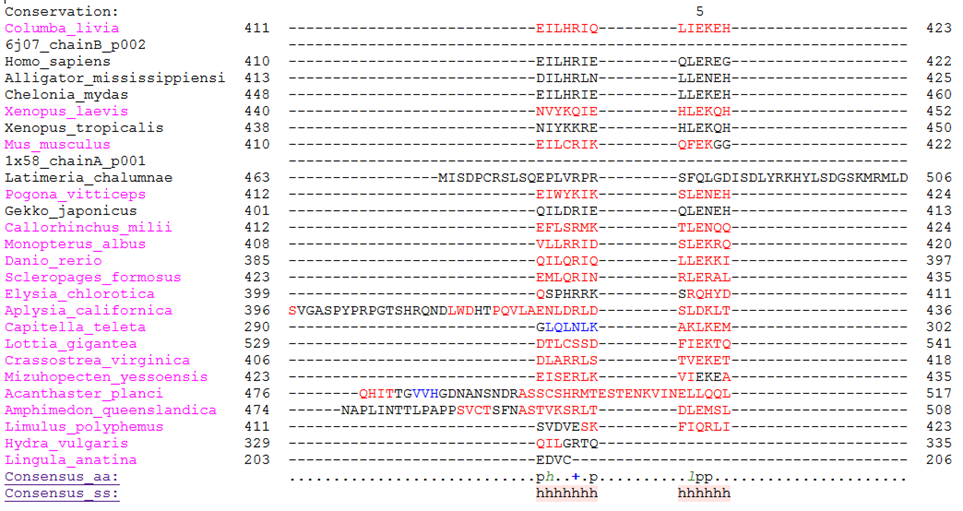** |
| **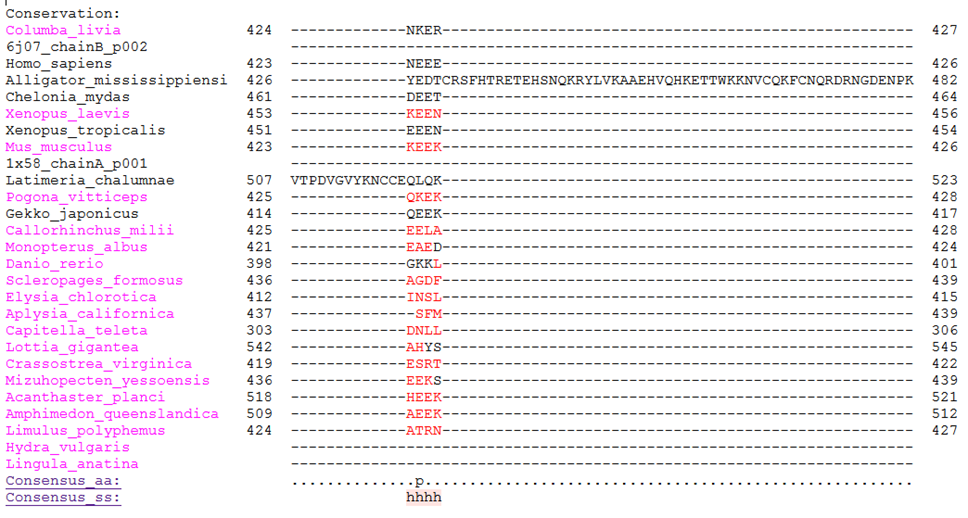** |
| **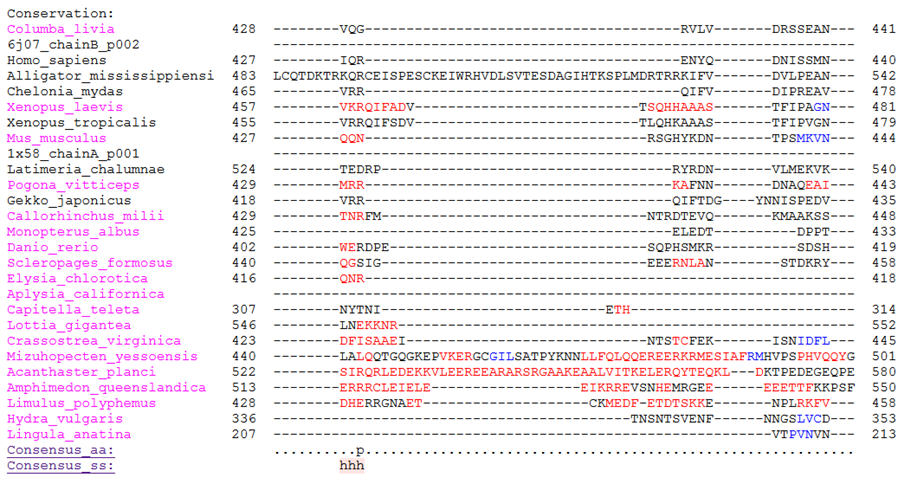** |
| **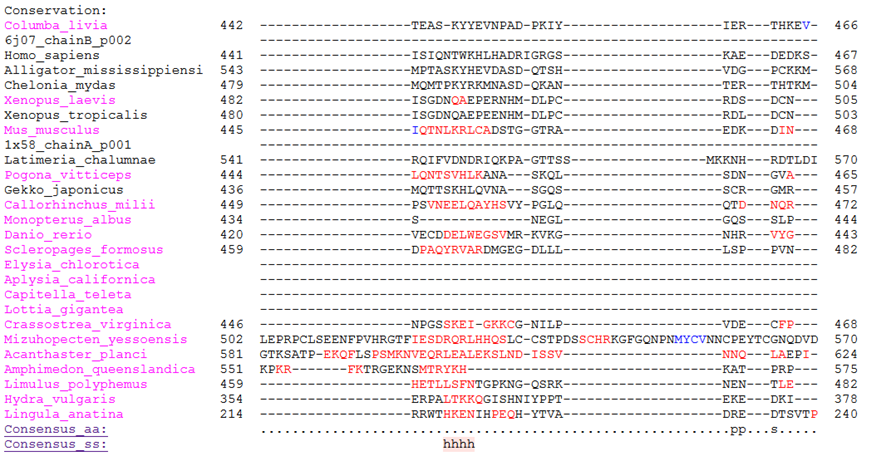** |
| **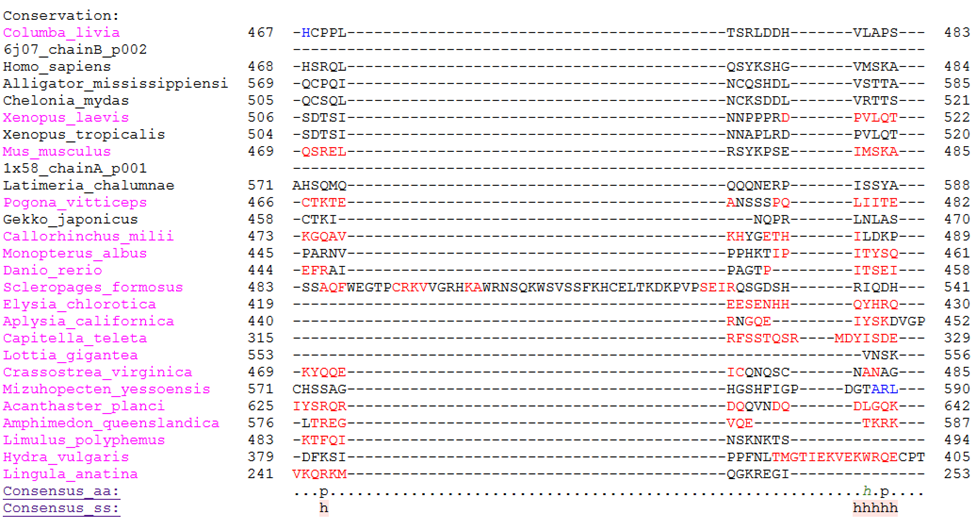** |
| **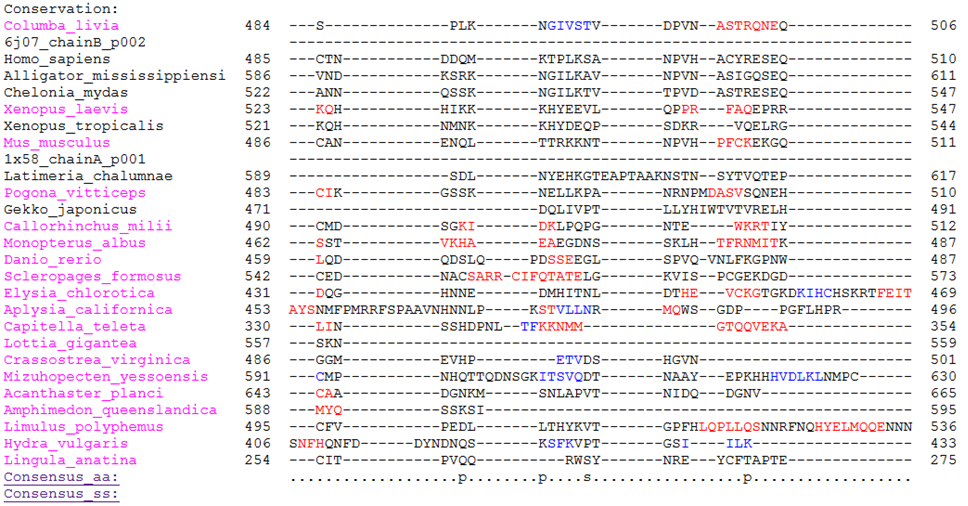** |
| **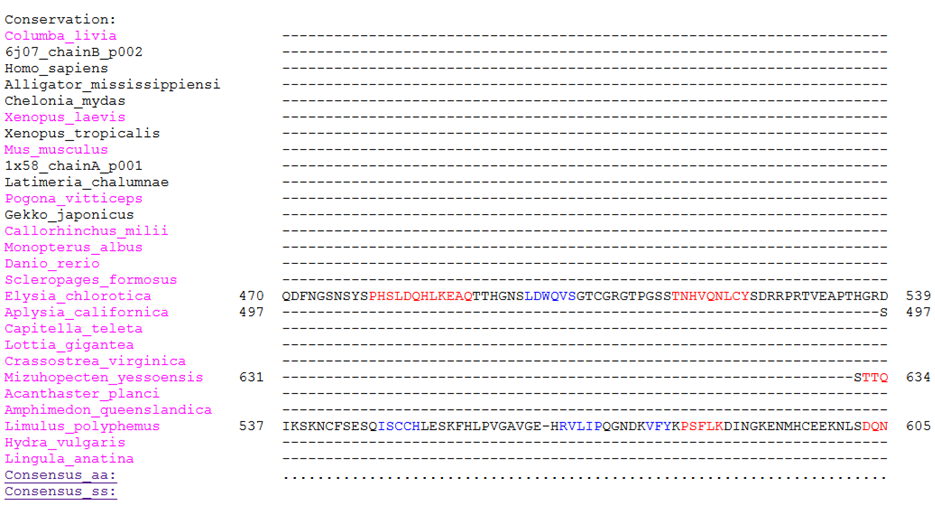** |
| **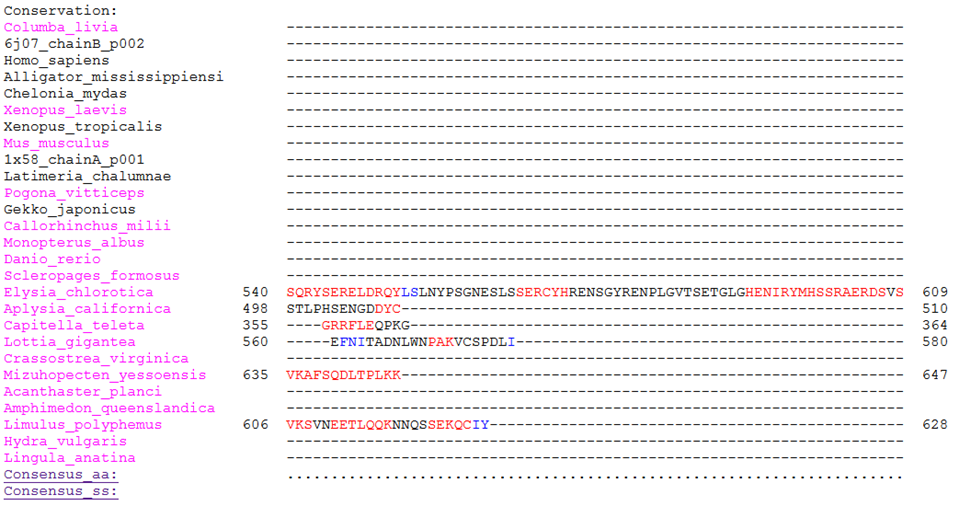** |
| **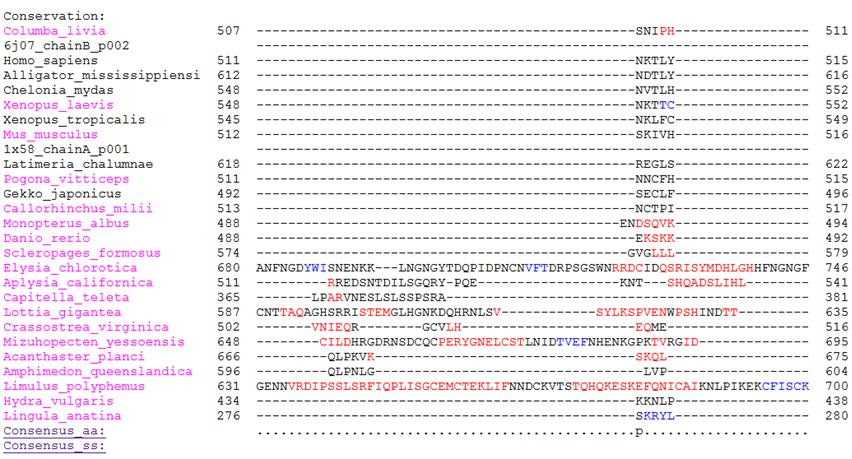** |
| **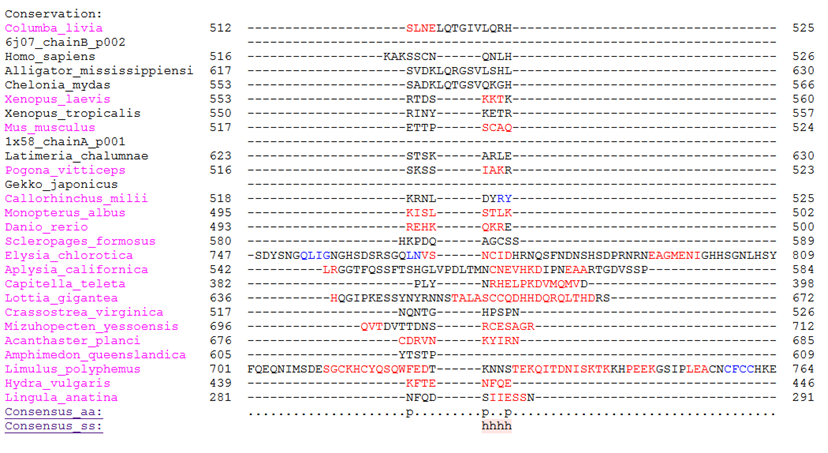** |
| **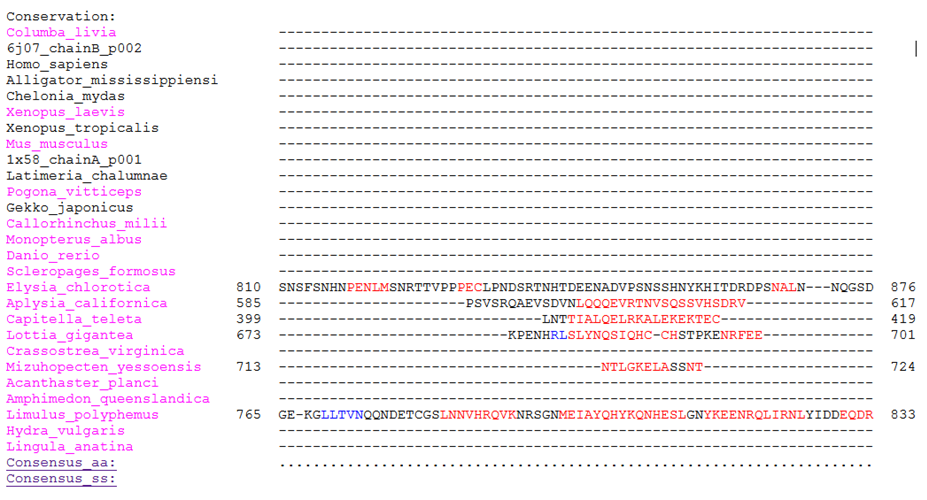** |
| **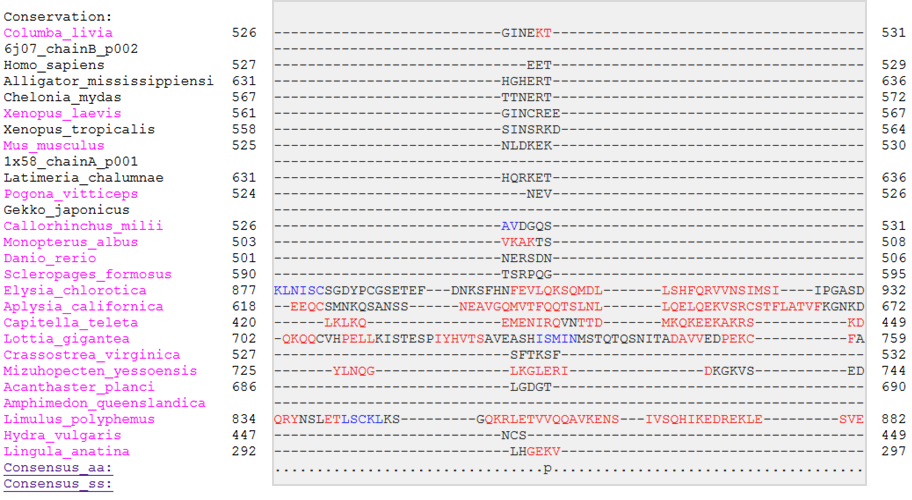** |
| **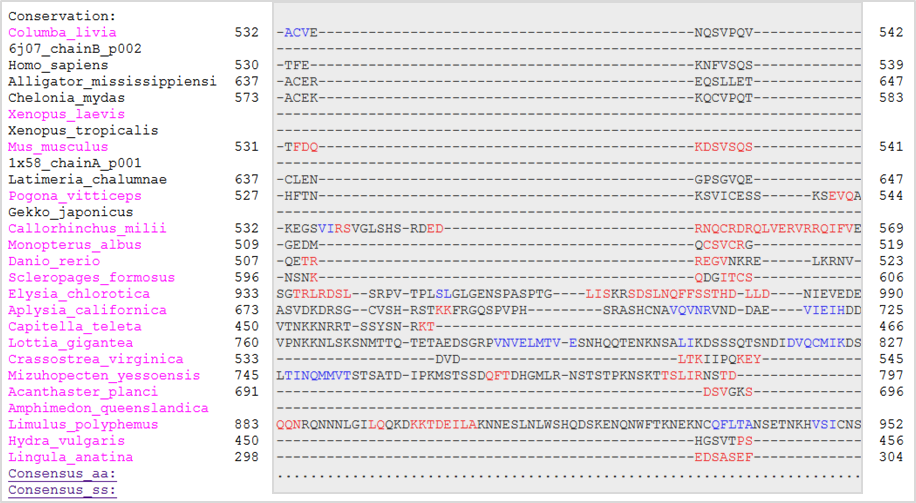** |
| **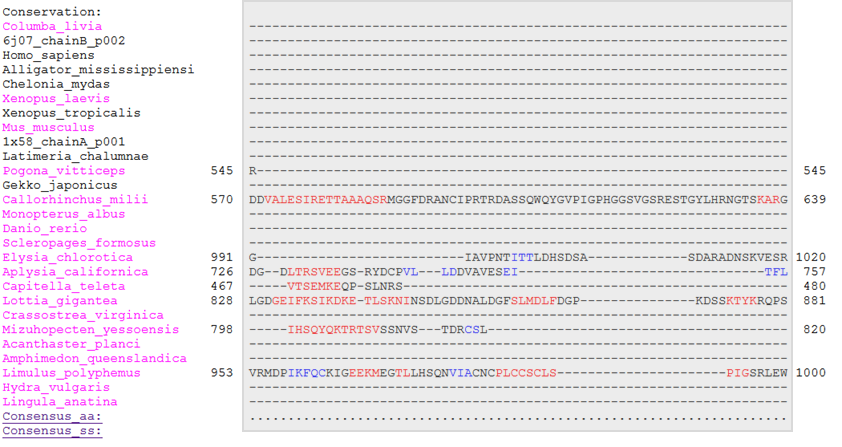** |
| **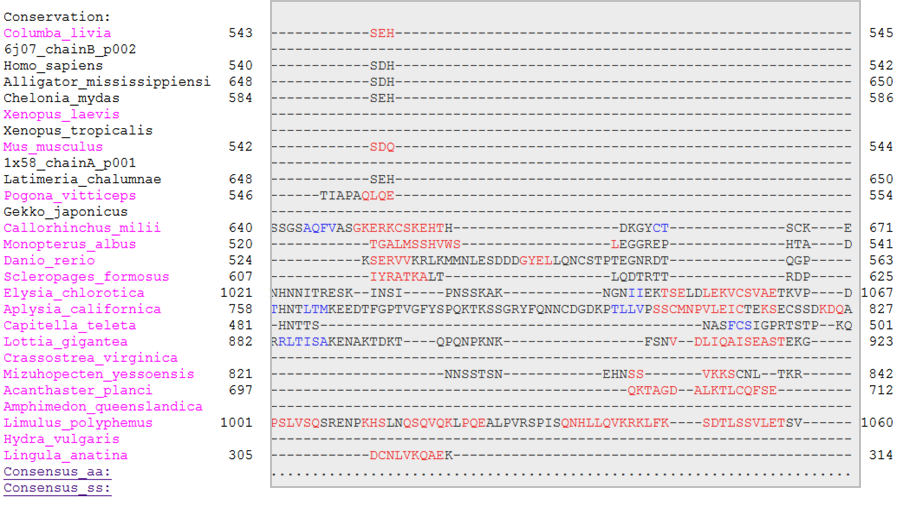** |
| **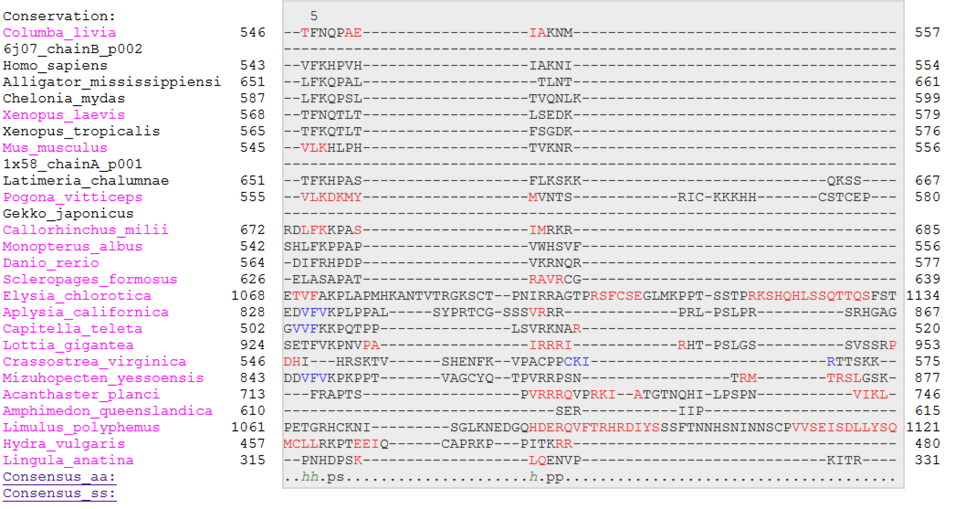** |
| **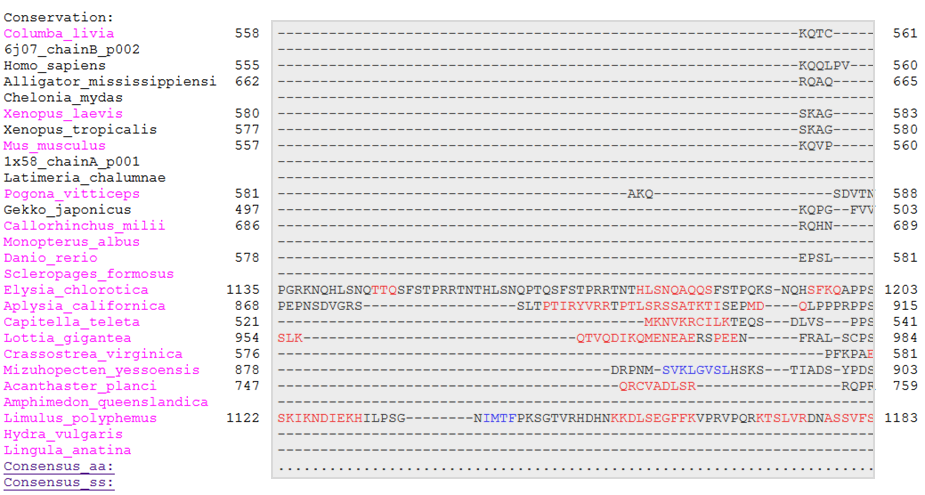** |
| **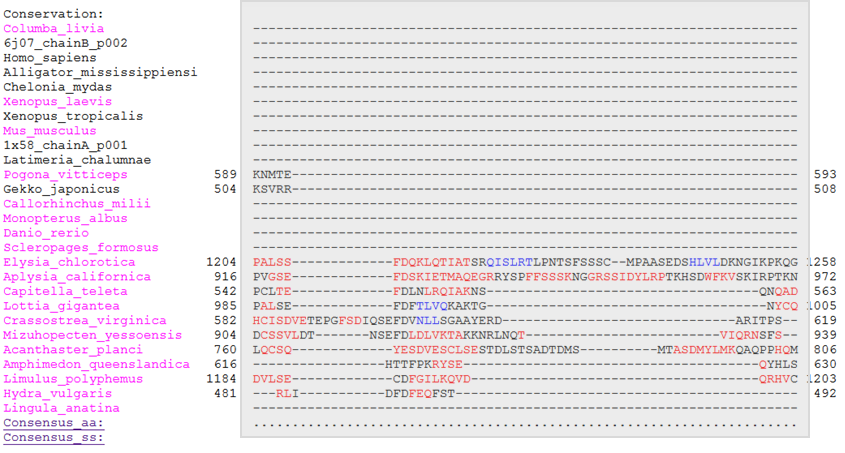** |
| **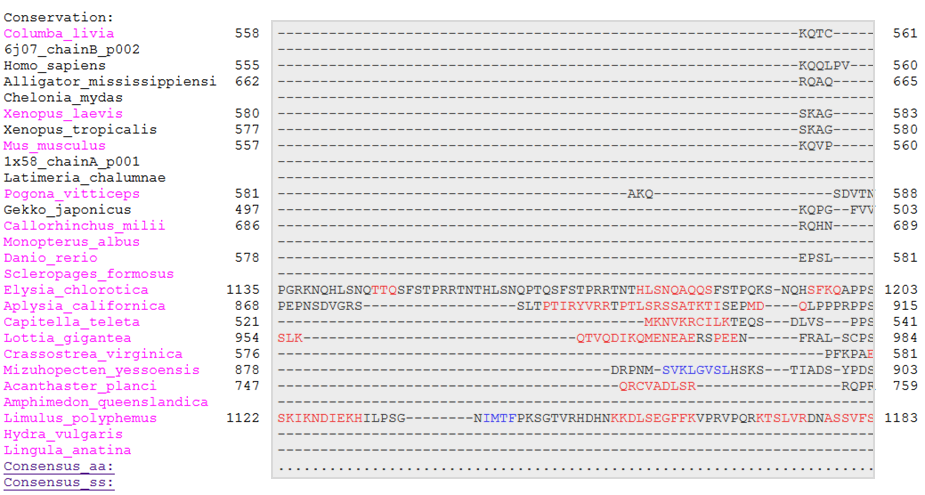** |
| **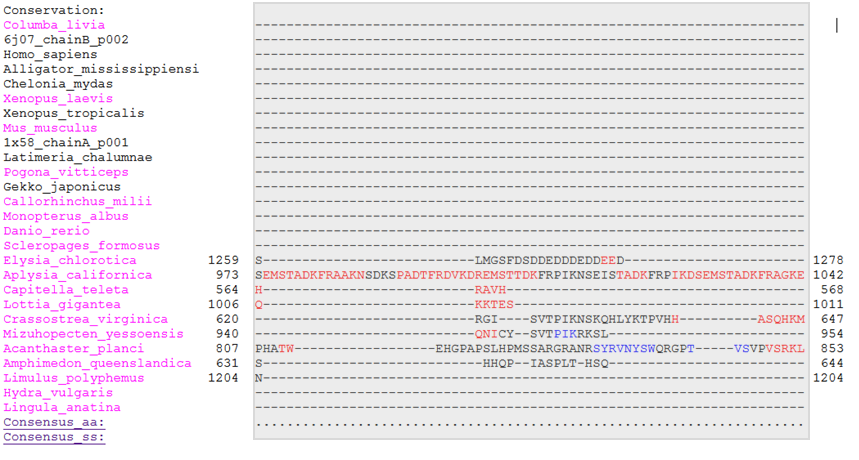** |
| **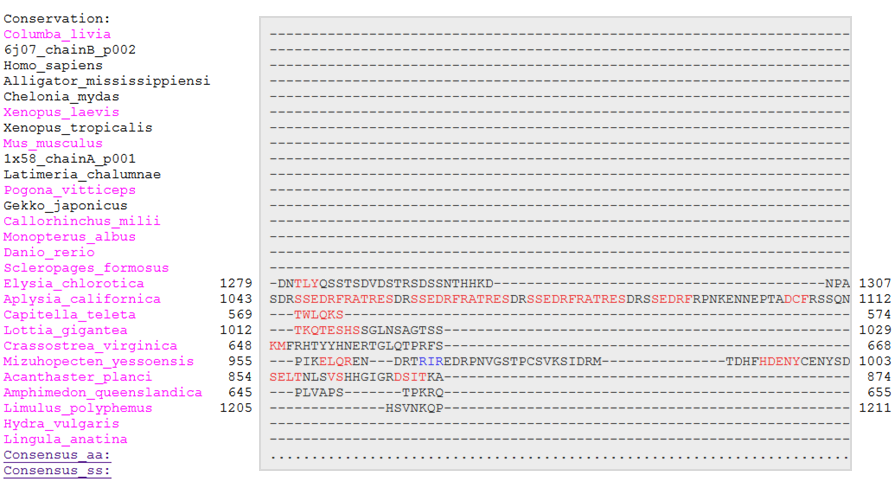** |
| **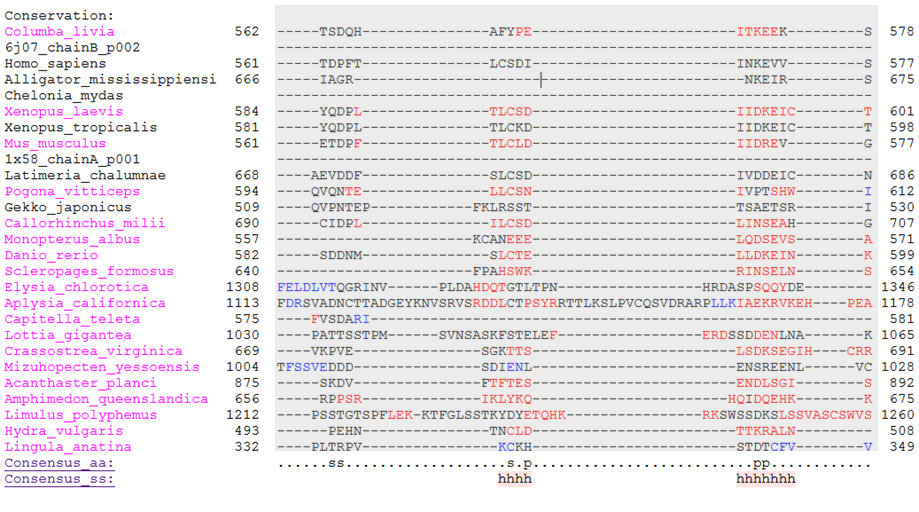** |
| **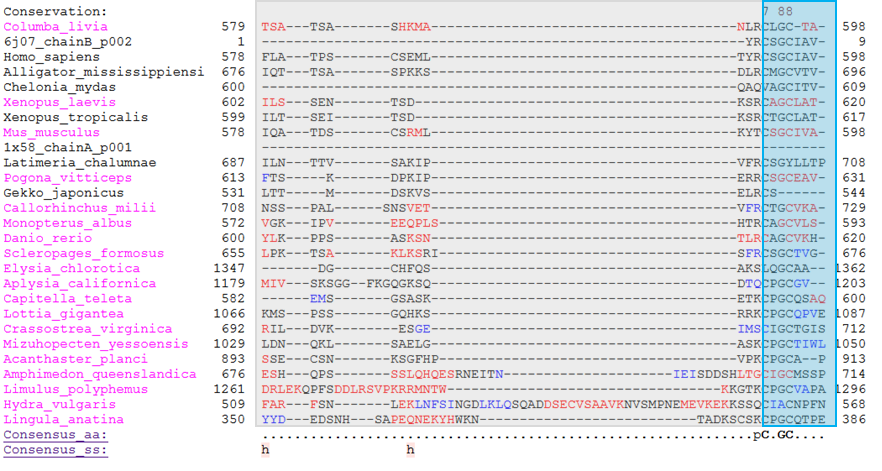** |
| **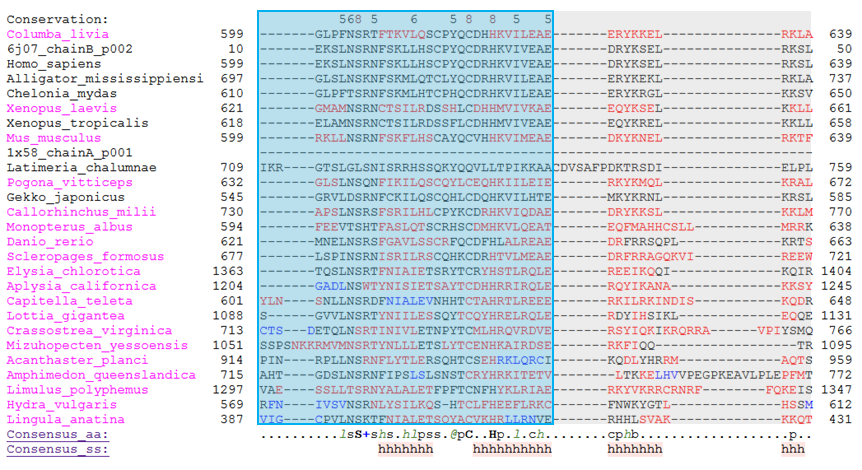** |
| **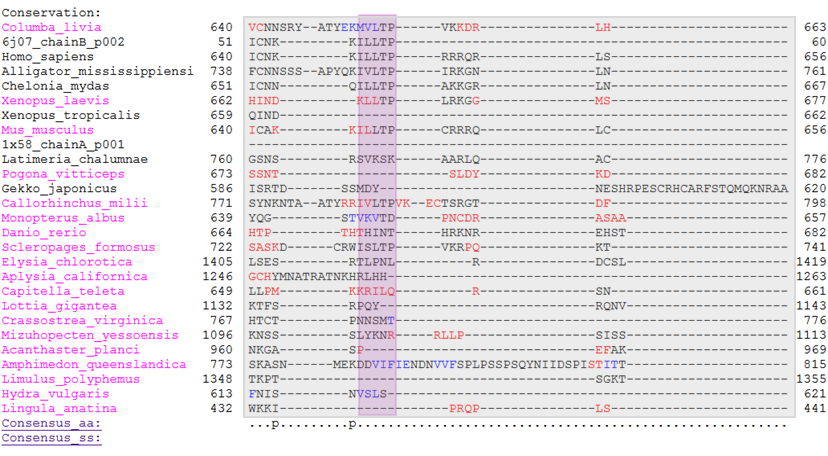** |
| **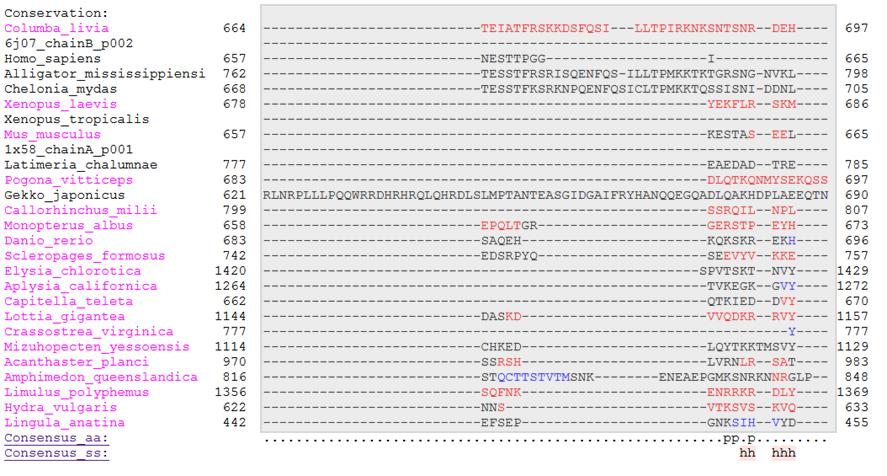** |
| **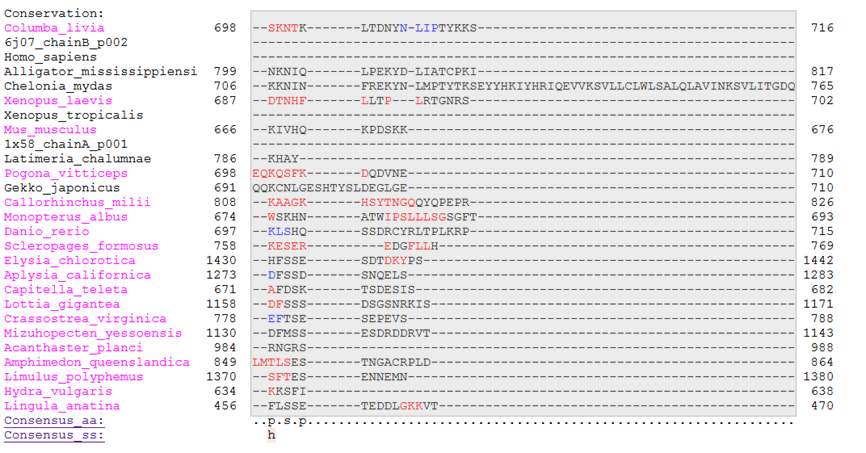** |
| **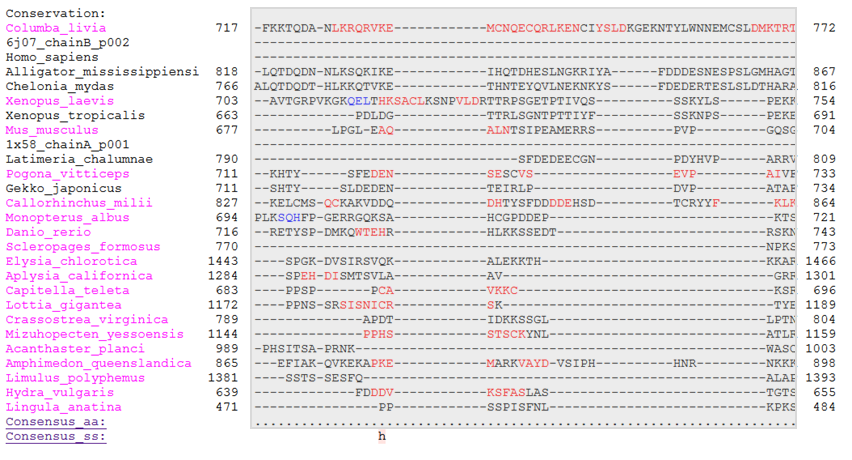** |
| **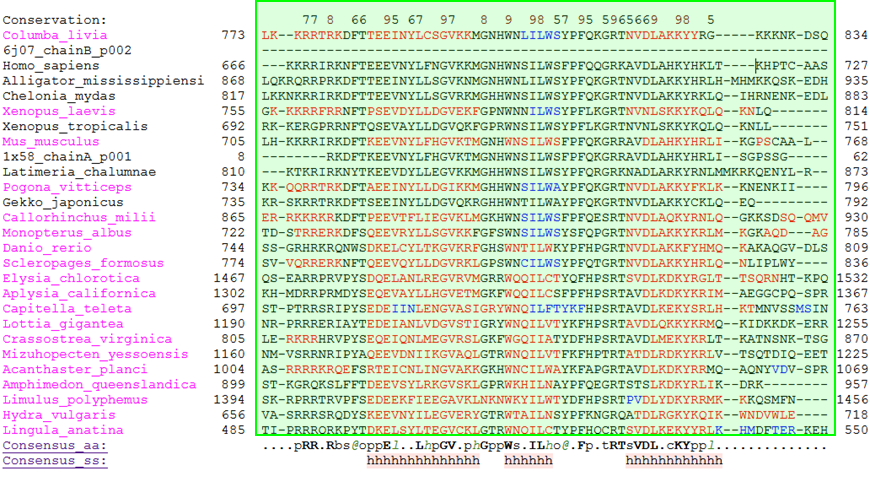** |

**Figure S1**.-Multiple sequence alignment of candidate TERB1 sequences by PROMALS3D. The sequences with magenta names are colored according to their predicted secondary structures (red: alpha-helix; blue: beta-strand). The sequences with black names belong to the same taxonomic groups of the nearest magenta sequence above them in the alignment. The first line in each block shows conservation indices for positions with a conservation index above 4. The highlighted rectangles in the alignment indicates domain/motif boundaries in mouse TERB1 protein. Each color represents a domain: ARM repeat domain (orange), TRFB domain (grey), T2B; binding site of TERB2 (light blue), TRF1-binding motif (violet) and MYB domain (green).

| **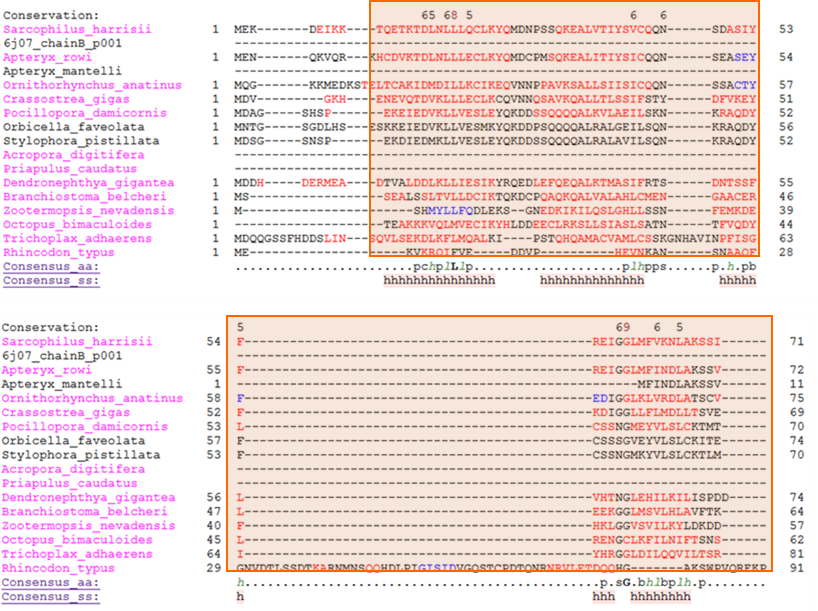** |
| --- |
| **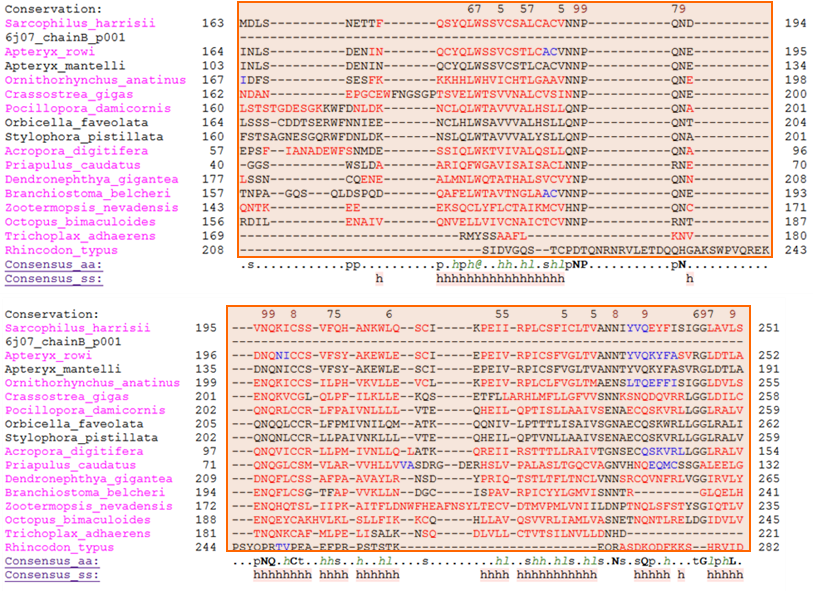** |
| **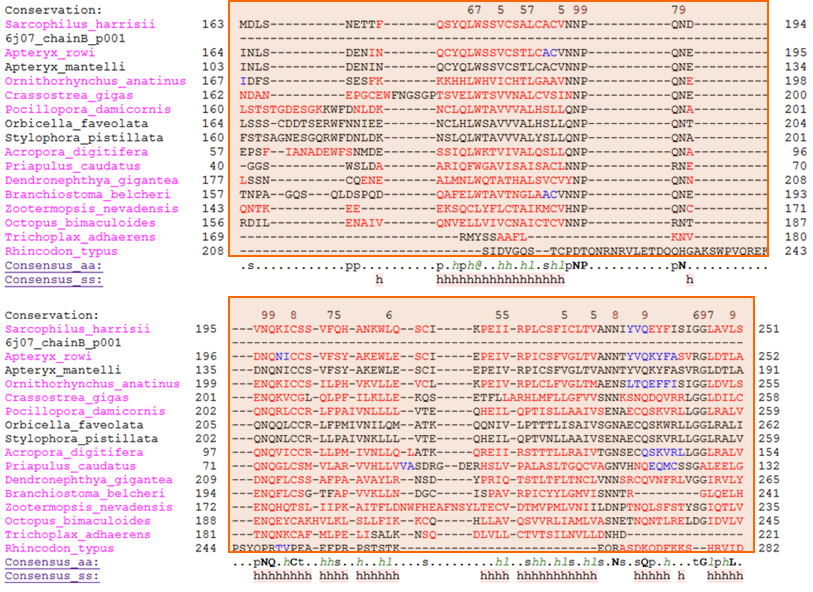** |
| **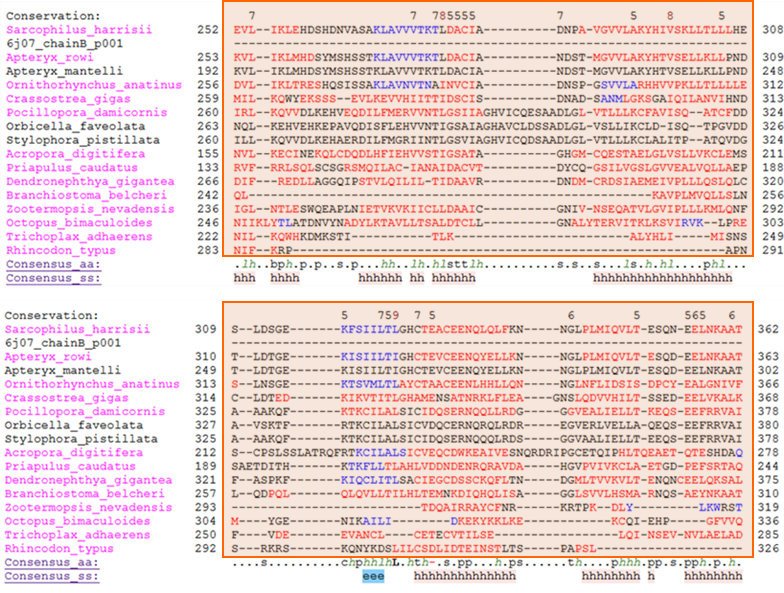** |
| **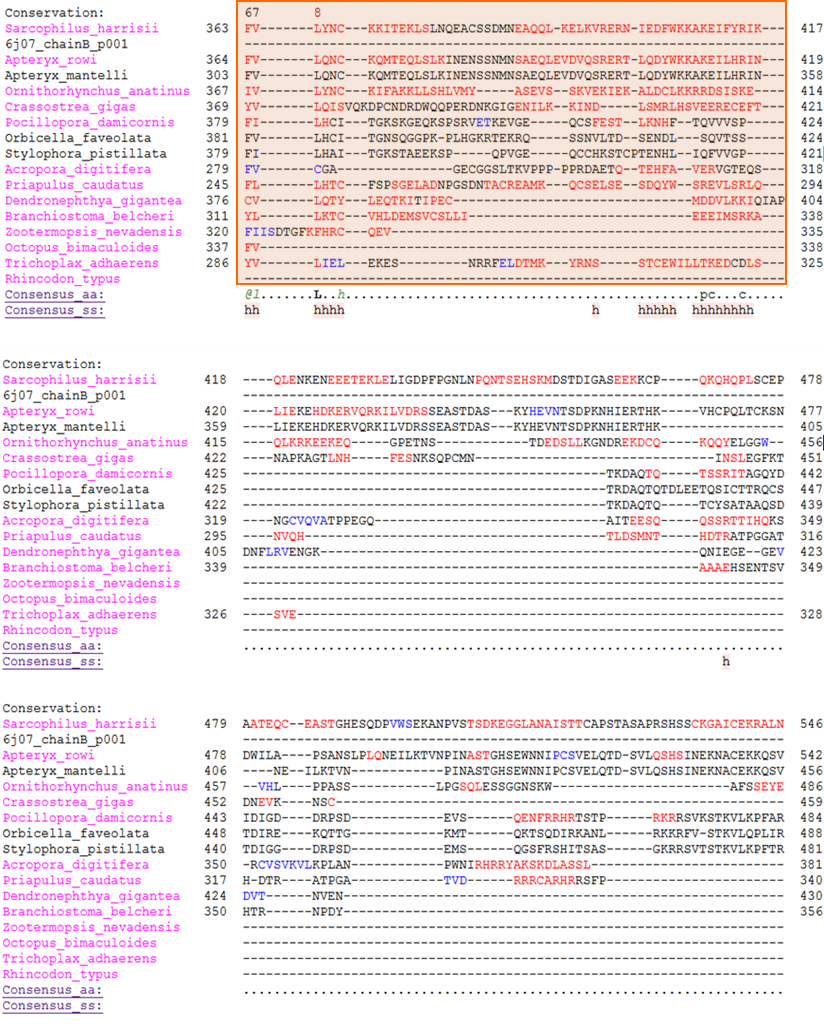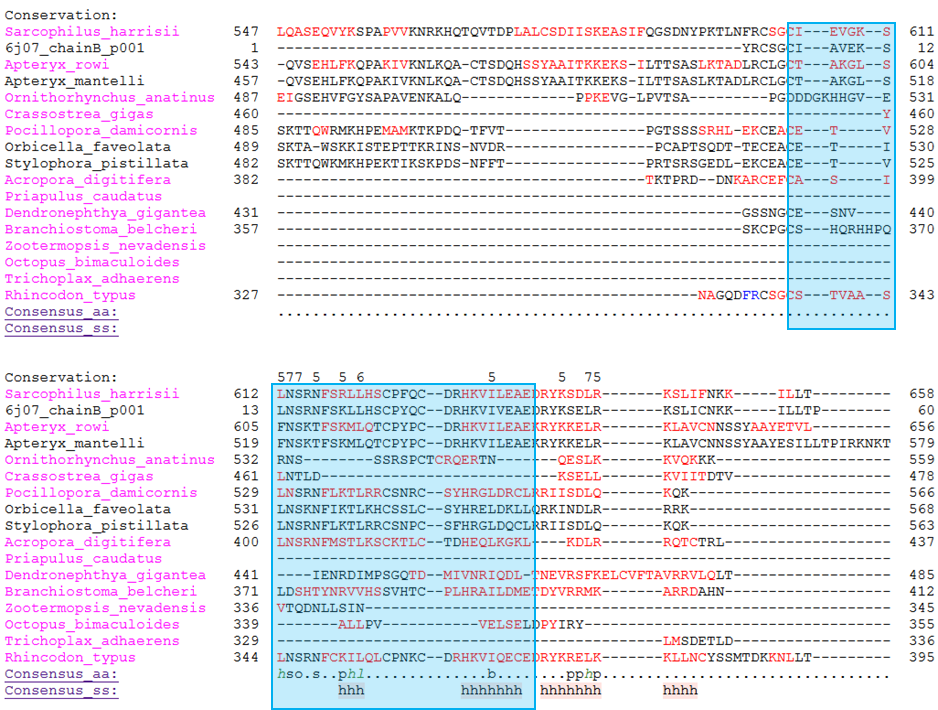** |
| **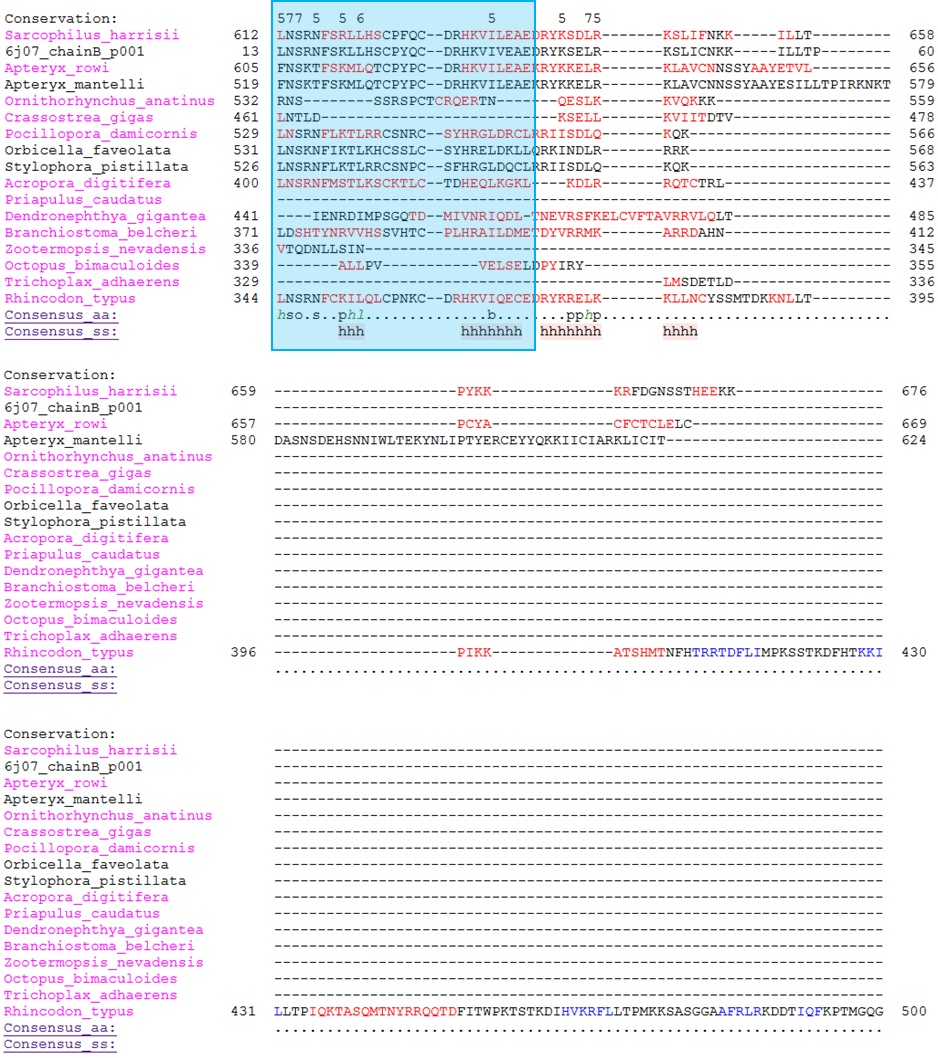** |

**Figure S2**.-Multiple sequence alignment of candidate TERB1 sequences that lack a MYB-domain by Promals3D. The sequences with magenta names are colored according to predicted secondary structures (red: alpha-helix, blue: beta-strand). The sequences with black names belong to the same taxonomic group of the nearest magenta sequence above them. The first line in each block shows conservation indices for positions with a conservation index above 4. The highlighted rectangles in the alignment correspond to the mouse TERB1 N-terminal ARM repeat domain (orange) and TERB2-binding site in TERB1 (light blue).

| 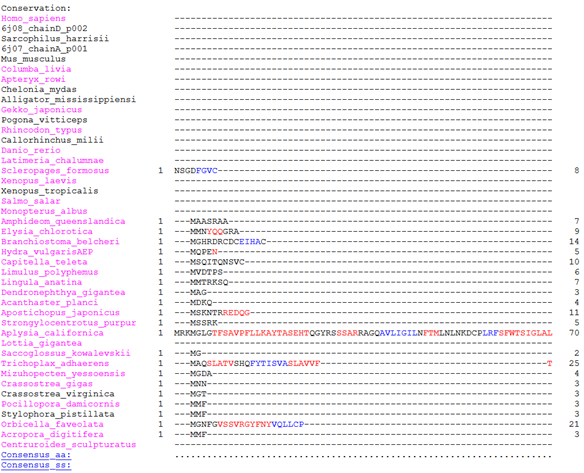 |
| --- |
| 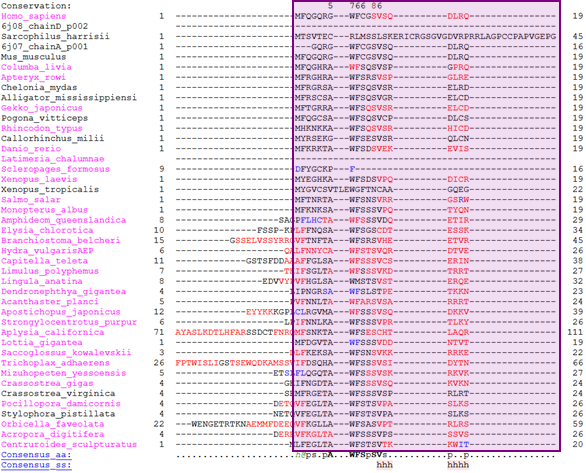 |
| 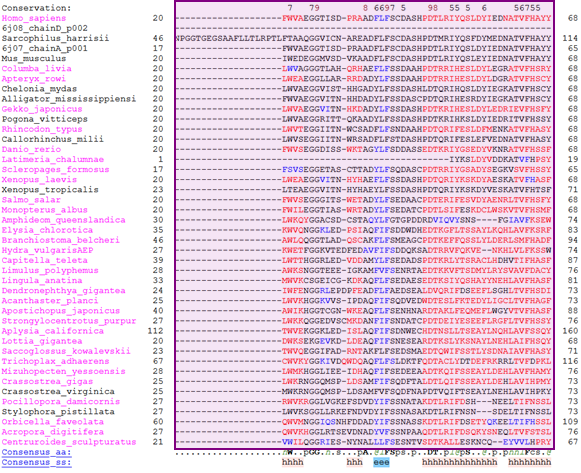 |
| 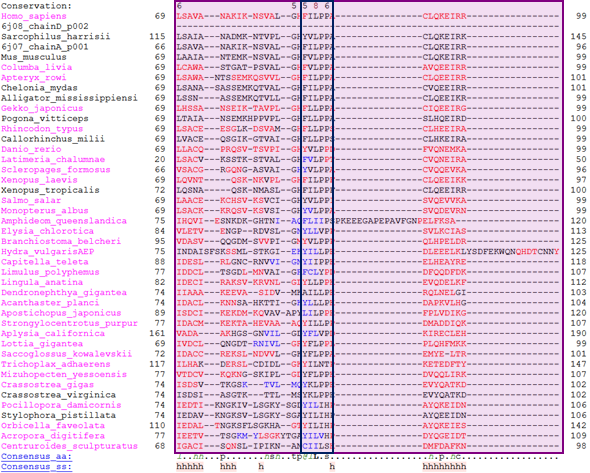 |
| 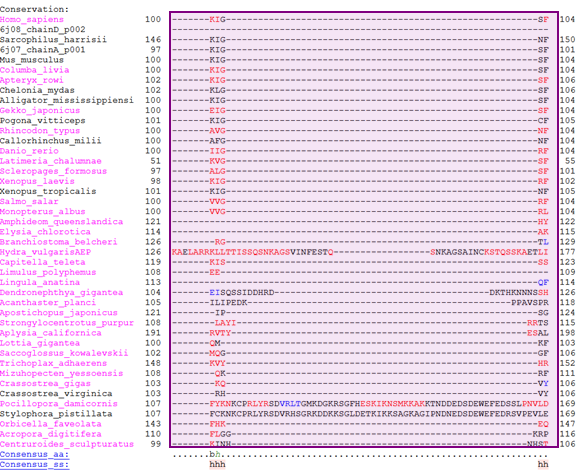 |
| 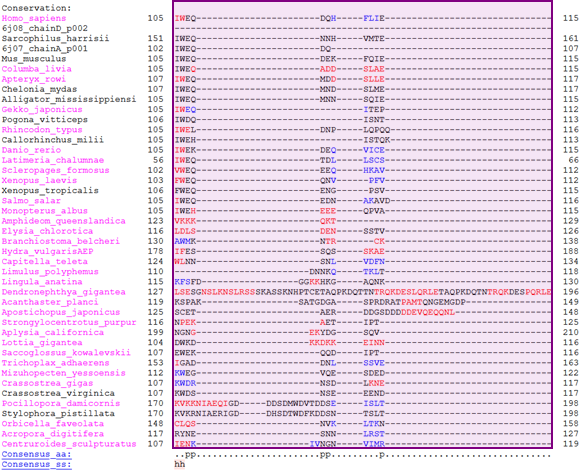 |
| 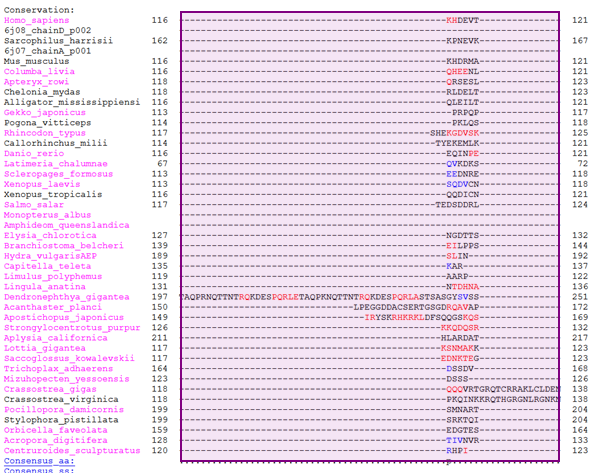 |
| 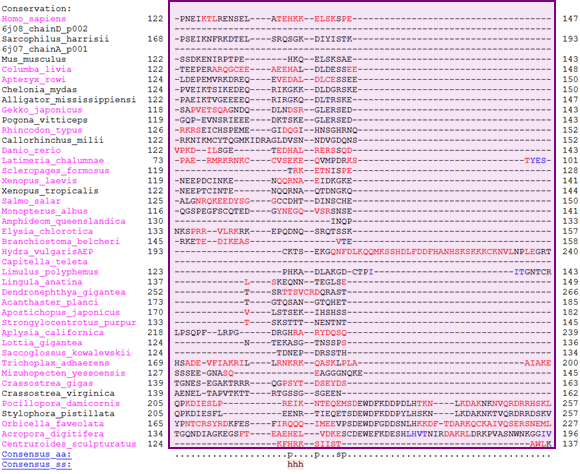 |
| 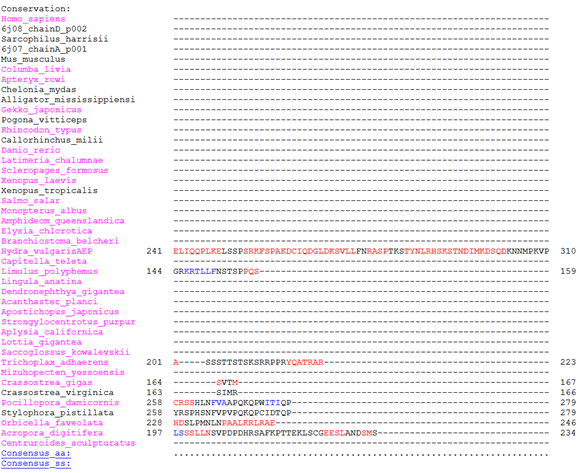 |
| 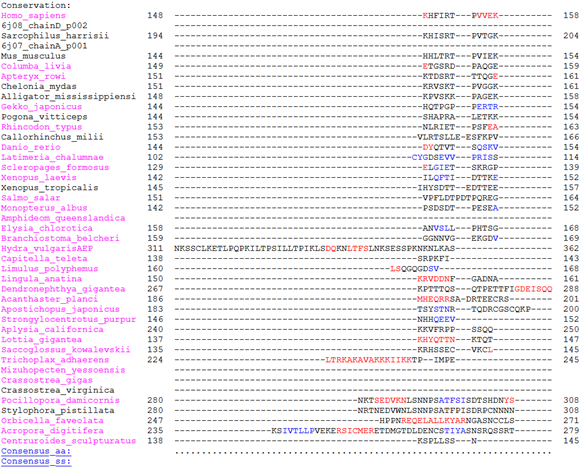 |
| 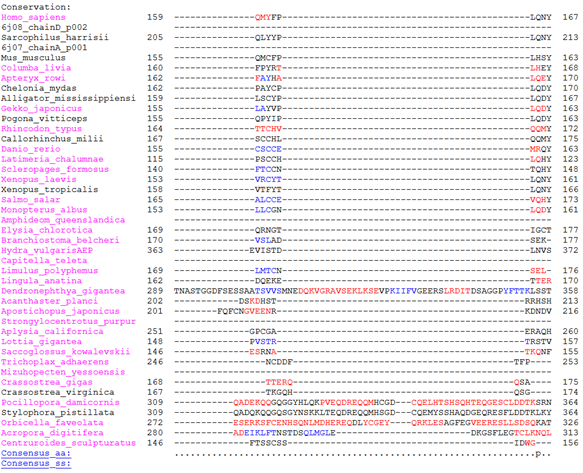 |
| 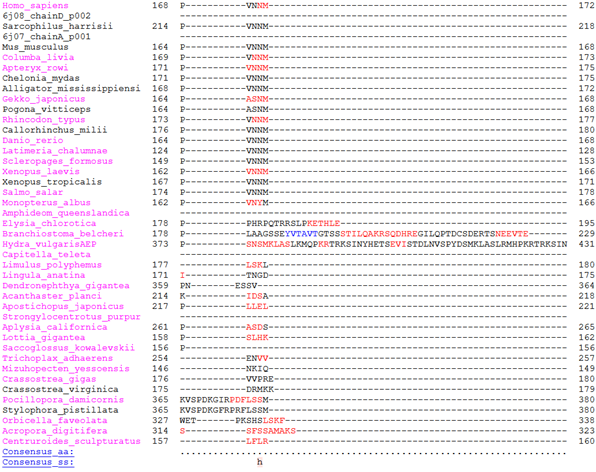 |
| 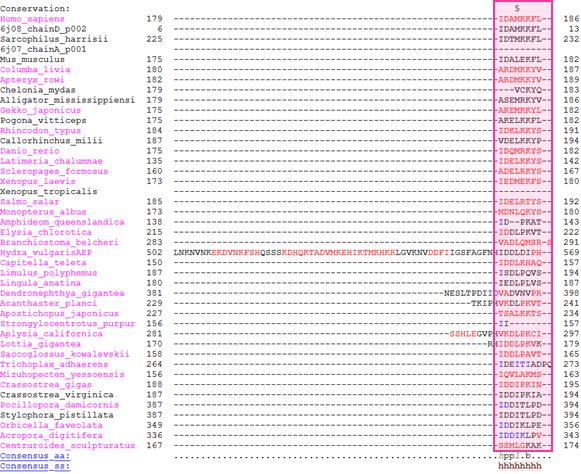 |
| 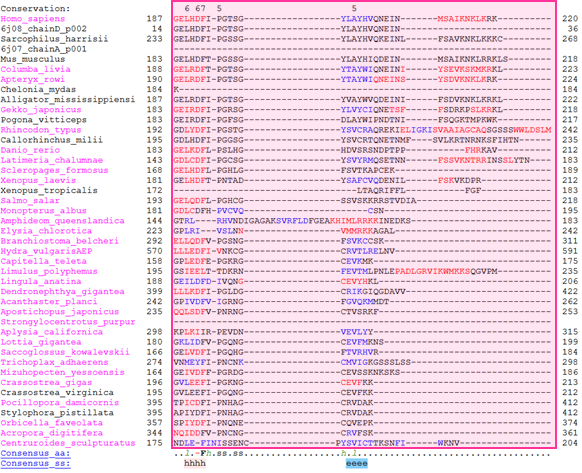 |

**Figure S3**.- Multiple sequence alignment of TERB2 sequences with PROMALS3D. The sequences with magenta names are colored according to predicted secondary structures (red: alpha-helix, blue: beta-strand). The sequences with black names belong to the same taxonomic group as the nearest magenta sequence above them. The first line in each block shows conservation indices for positions with a conservation index above 4. The highlighted rectangles in the alignment correspond to the mouse TERB2 N-terminal domain (violet) and MAJIN-binding site (pink). The highly conserved motif [F/YxLxP] detected in all TERB2 N-terminal sequences is shown in the dark blue rectangle.

| 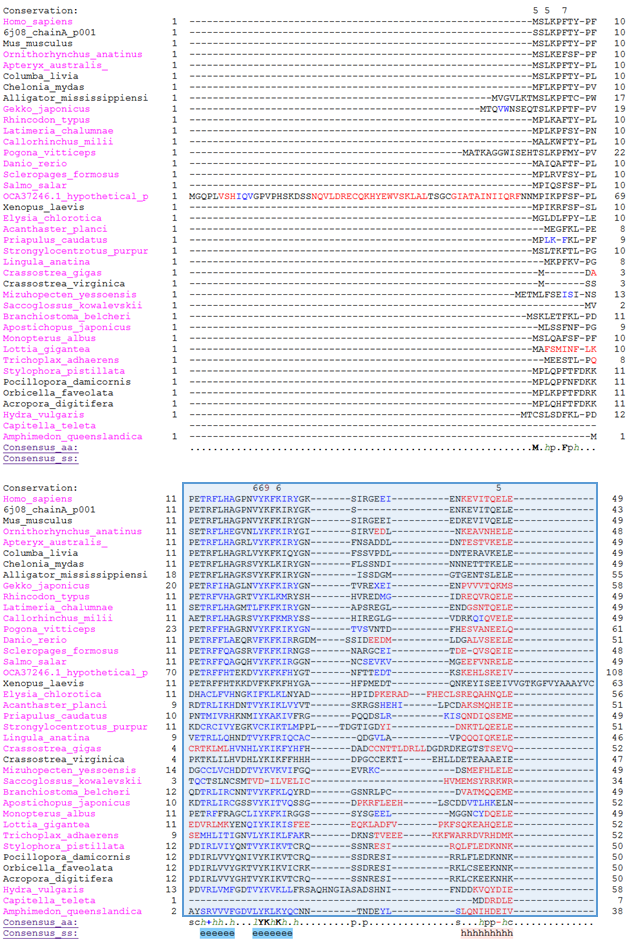 |
| --- |
| 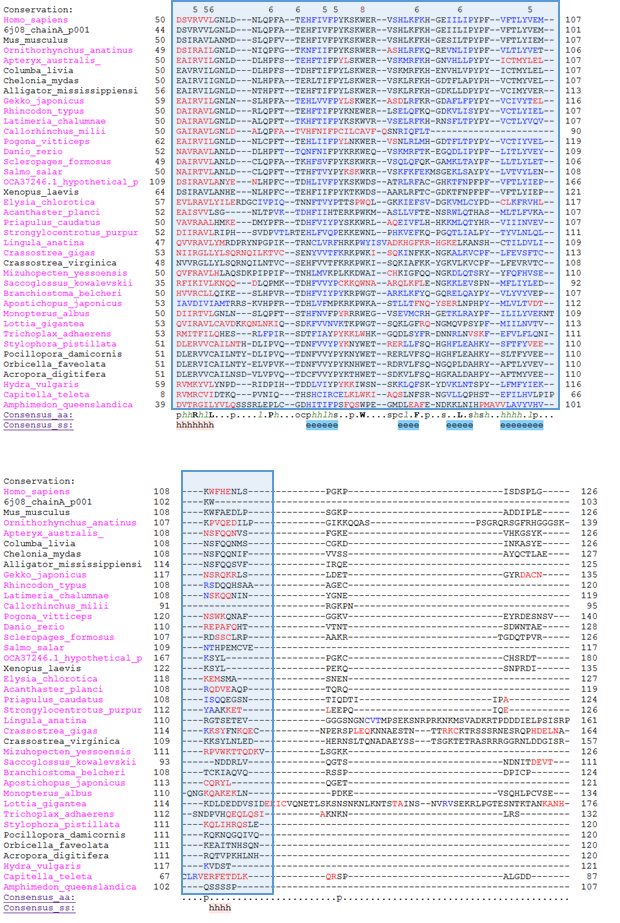 |
| 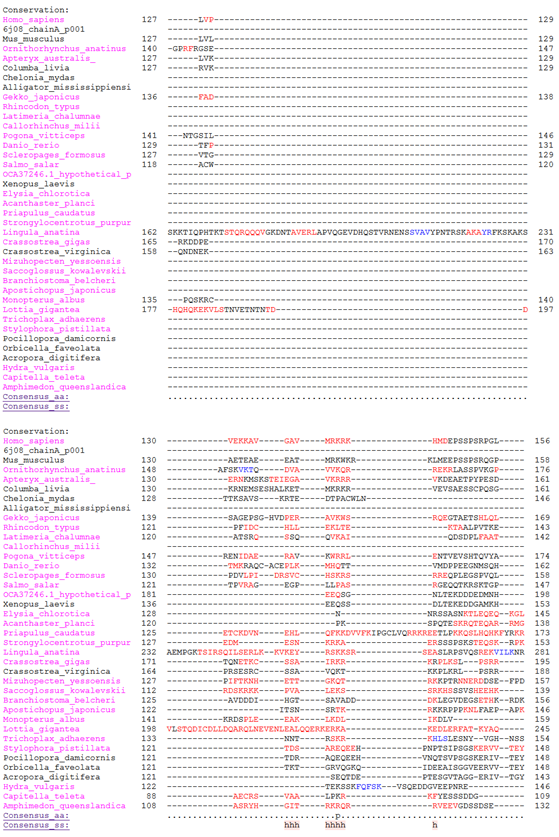 |
| 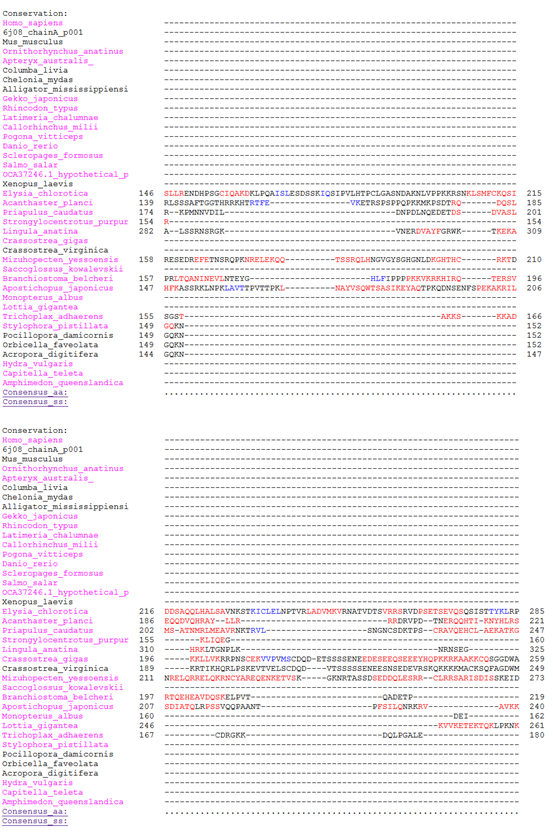 |
| 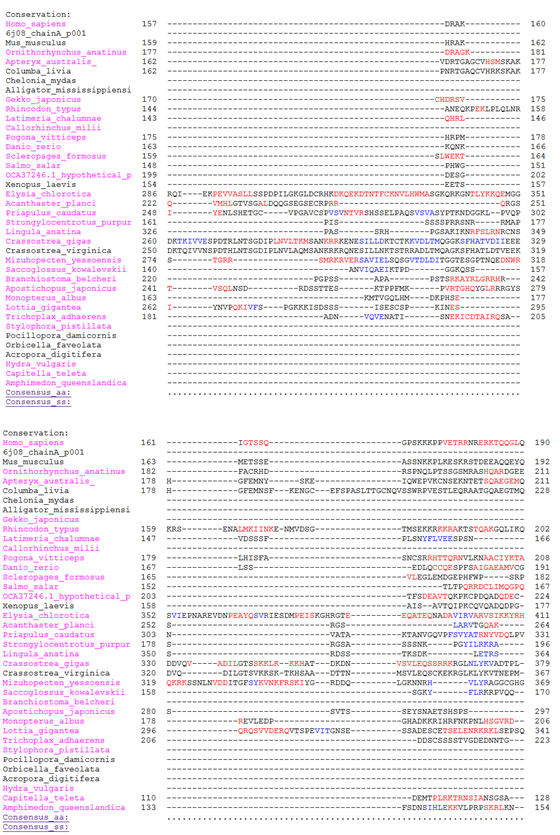 |
| 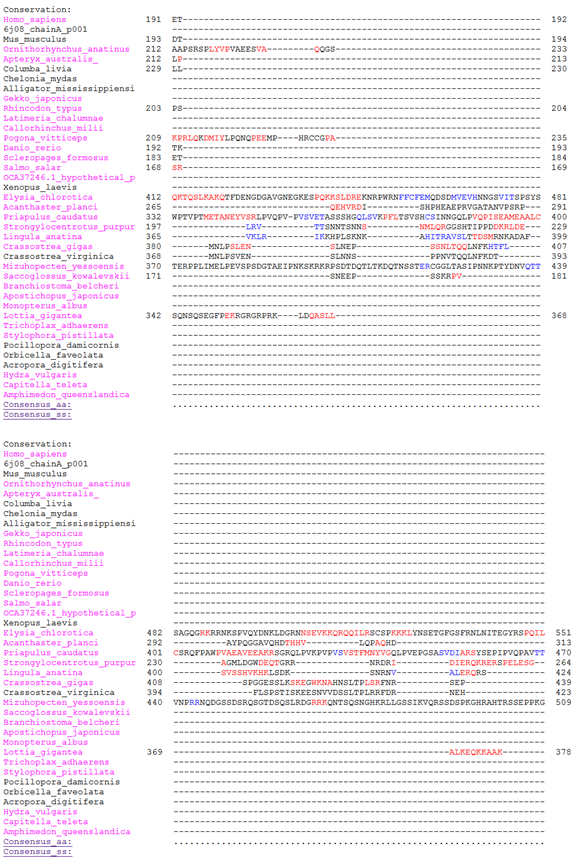 |
| 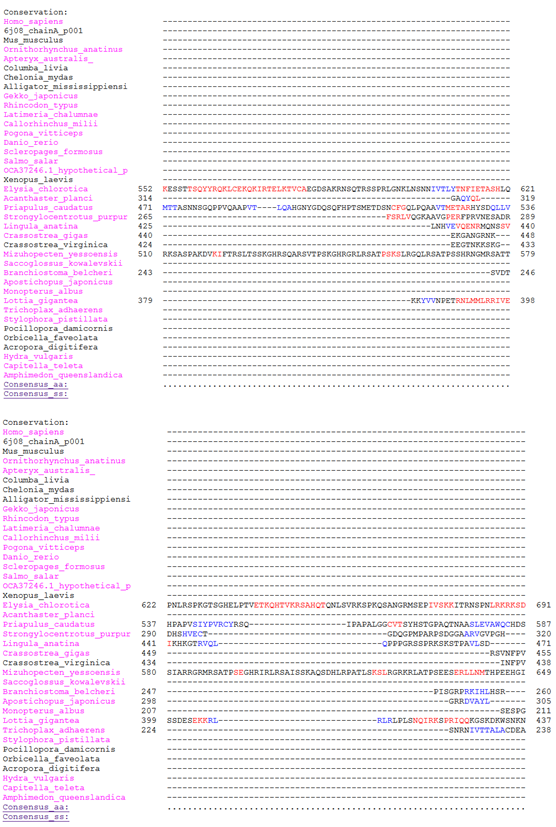 |
| 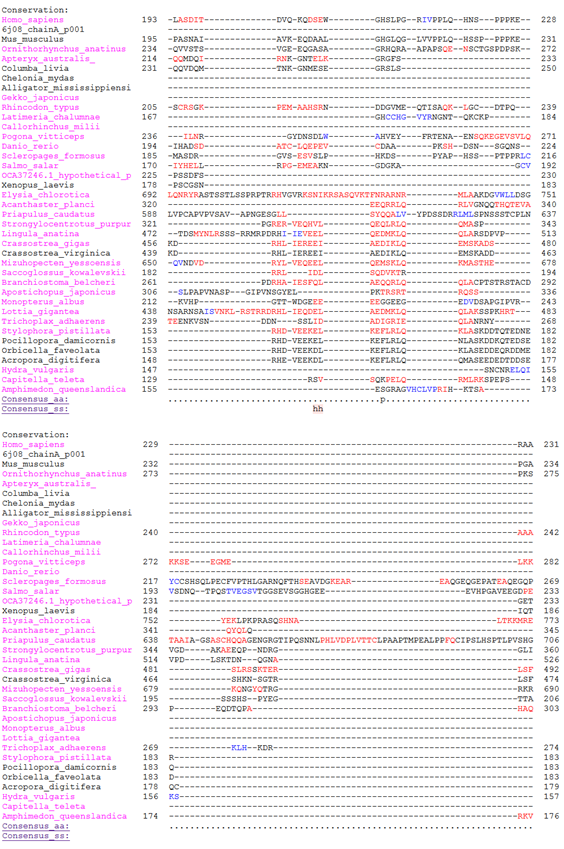 |
| 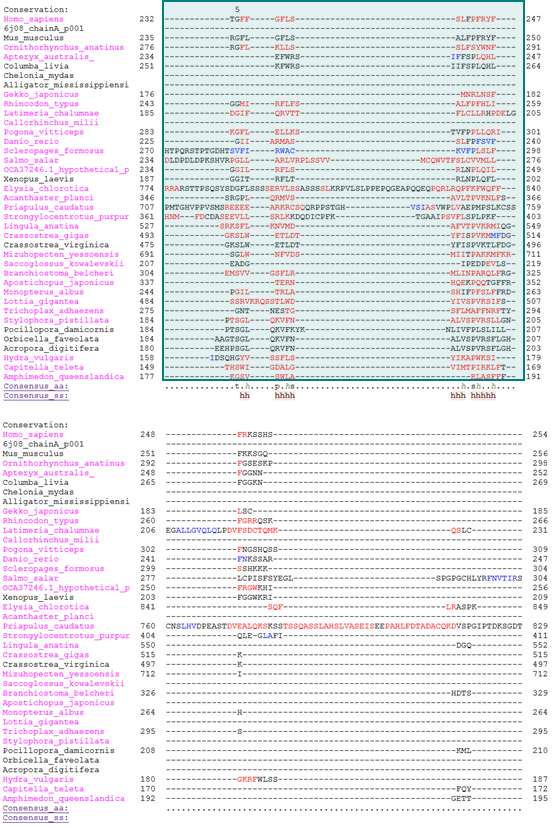 |

**Figure S4**.-Multiple sequence alignment of MAJIN sequences carried out using the PROMALS3D. The sequences with magenta names are colored according to predicted secondary structures (red: alpha-helix, blue: beta-strand). The sequences with black names belong to the same taxonomic group of the nearest magenta sequence above. The first line in each block shows conservation indices for positions with a conservation index above 4. The highlighted rectangles in the alignment correspond to the mouse MAJIN N-terminal domain (blue) and TM domain (in dark green).

**Table S5.-**Primers used in this study.

| Application | Primer Sequence | Fragment length | | | Annealing Temperature |
| --- | --- | --- | --- | --- | --- |
| Cloning full-length cDNA | | | | | |
| *Hydra vulgaris* AEP Terb1 | HyTerb1_FL_Fwd  5’ TCAATCATATCCAAACCAACAA 3’ | | 2380 bp | 61 °C | |
|  | Hy_Terb1_FL_Rev  5’ ATTTCTCGCTTTTATTCATTGTCA 3’ | |  |  |  |
| *Hydra vulgaris* AEP Terb2 | HyTerb2_FL_Fwd  5’ ATGATGCAGCCAGAAAACCA 3’ | | 1780 bp | 60 °C | |
|  | Hy_Terb2_FL_Rev  5’ TCAAACATTCAATTCACGCA 3’ | |  |  |  |
| *Hydra vulgaris* AEP Majin | Hy_MAJIN_FL_Fwd  5’ CATGACGTGTAGTTTGTCTGATT 3’ | | 699 bp | 63 °C | |
|  | Hy_MAJIN_FL_Rev  5’ TGTCTGACCGACCGGATC 3’ | |  |  |  |
| RT-PCR | | | | | |
| *Hydra vulgaris* AEP Terb1 | HyTerb1_RT_Fwd  5’ TTTTCTACGCCTGAGCACAAT 3’ | | 497 bp | 63°C | |
|  | HyTerb1_RT_Rev  5’ GAGGTTCCAGTCGAAGCAAG 3’ | |  |  |  |
| *Hydra vulgaris* AEP Terb2 | HyTerb2_RT_Fwd  5’ TGACTTCGAAAAATGGCAGA 3’ | | 447 bp | 64°C | |
|  | HyTerb2_RT_Rev  5’ CCATCAAGAAAGTTCTCGCC 3’ | |  |  |  |
| *Hydra vulgaris* AEP Majin | HyMajin_RT_Fwd  5’ CATGACGTGTAGTTTGTCTGATT 3’ | | 369 bp | 60 °C | |
|  | HyMajin_RT_Rev  5’ TCAGTGGTGCTATCAACTTTTTTC 3’ | |  |  |  |
| *Hydra vulgaris* AEP Actin | HyActin_Fwd  5’ AGGAGTCATGGTTGGTATGGGA 3’ | | 448 bp | 63 °C | |
|  | HyActin_Rev  5’ AATCTCGTCCTGCTAAATCCA 3’ | |  |  |  |
| Whole Mount In Situ Hybridization | | | | | |
| *Hydra vulgaris* AEP TERB1 | HyTerb1_WMIH_Fwd  5’ TTTTCTACGCCTGAGCACAAT 3’ | | 649 bp | 67 °C | |
|  | HyTerb1_WMIH_Rev  5’ TTCCACGTAAATCTGTTGCTTG 3’ | |  |  |  |
| *Hydra vulgaris* AEP TERB2 | HyTerb2_WMIH_Fwd  5’ TGACTTCGAAAAATGGCAGA 3’ | | 447 bp | 64°C | |
|  | HyTerb2_WMIH_Rev  5’ CCATCAAGAAAGTTCTCGCC 3’ | |  |  |  |
| *Hydra vulgaris* AEP MAJIN | HyMajin_WMIH_Fwd  5’ CATGACGTGTAGTTTGTCTGATT 3’ | | 699 bp | 63 °C | |
|  | HyMajin_WMIH_Rev  5’ TGTCTGACCGACCGGATC 3’ | |  |  |  |
| *Hydra vulgaris* AEP SYCP3 | HySycp3_Fwd  5’ GTCCGCAATTAGTGCAGCAATGAACGA 3’ | | 714 bp | 64°C | |
|  | HySycp3_Rev  5’GACTTAAACACTGTGTAGCAAGCTTTGAAGCGA 3’ | |  |  |  |

| 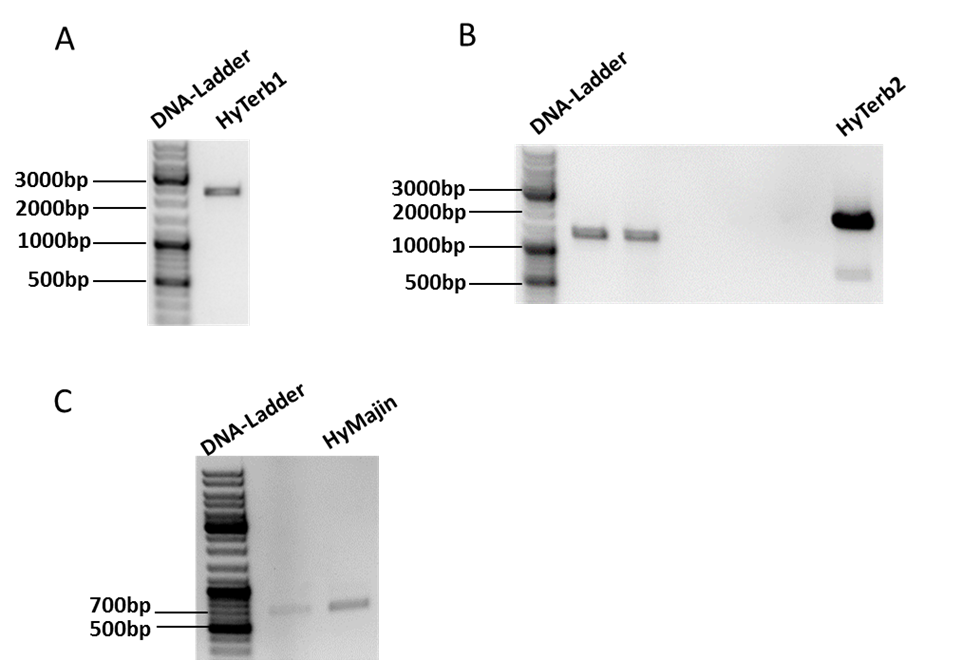 |
| --- |

**Figure S5**.-Identification of full-length cDNA of *Hydra* Terb1 (A), Terb2 (B) and Majin (C). The expected size of the coding region amplified is: 2380 bp (HyTerb1), 1780 bp (HyTerb2), and 699 bp (HyMajin).

| 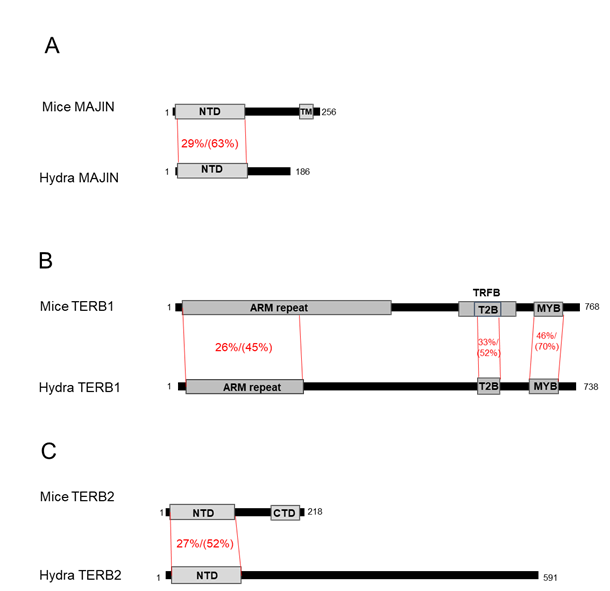 |
| --- |

**Figure S6.-**Schematic comparison of mouse and *Hydra* MAJIN (A), TERB1 (B) and TERB2 (C) proteins through blast. The mouse sequence accession IDs are: MAJIN (GenBank ID: NP_001159391), TERB1 (GenBank ID: NP_851289), TERB2 (GenBank ID: NP_083190). We used predicted translation product from our full-length cDNA experiments in *Hydra*. Sequence identity and similarity (in parenthesis) at the amino acid level are given (%). The gray boxes represent domains. NTD, N-terminal domain; TM, transmembrane domain; ARM repeats, armadillo repeats; T2B, binding region of TERB2 in TERB1 protein.
